# Nonsense Mutation in *USH2A* Exon-13 Activates the Innate Immune Response in Müller Glial Cells

**DOI:** 10.3390/ijms27041636

**Published:** 2026-02-07

**Authors:** Rossella Valenzano, Xuefei Lu, Andrew McDonald, Ioannis Moustakas, Roberta Menafra, Aat A. Mulder, Roman I. Koning, Susan L. Kloet, Jun Yang, Hailiang Mei, Jan Wijnholds

**Affiliations:** 1 Department of Ophthalmology, Leiden University Medical Center, Albinusdreef 2, 2333 ZA Leiden, The Netherlands; r.valenzano@lumc.nl (R.V.); x.lu@lumc.nl (X.L.); a.mcdonald@lumc.nl (A.M.); 2 Department of Biomedical Data Sciences, Sequencing Analysis Support Core, Leiden University Medical Center, Albinusdreef 2, 2333 ZA Leiden, The Netherlands; i.moustakas@lumc.nl (I.M.); h.mei@lumc.nl (H.M.); 3 Department of Human Genetics, Leiden University Medical Center, Albinusdreef 2, 2333 ZA Leiden, The Netherlands; r.menafra@lumc.nl (R.M.); s.l.kloet@lumc.nl (S.L.K.); 4 Department of Cell and Chemical Biology, Electron Microscopy Facility, Leiden University Medical Center, Albinusdreef 2, 2333 ZA Leiden, The Netherlands; a.a.mulder@lumc.nl (A.A.M.); r.i.koning@lumc.nl (R.I.K.); 5 Department of Ophthalmology and Visual Sciences, John A. Moran Eye Center, University of Utah, 65 Mario Capecchi Drive, Salt Lake City, UT 84132, USA; jun.yang@hsc.utah.edu

**Keywords:** *USH2A*, *IFITM2*, *IFITM3*, retinal organoids, Müller glial cells, disrupted homeostasis, innate immune system, Usher syndrome

## Abstract

Pathological *USH2A* mutations cause Usher syndrome type II, characterized by progressive retinitis pigmentosa and hearing and balance impairment. This study aims to investigate the cellular mechanisms underlying *USH2A*-related retinal degeneration using human induced pluripotent stem cell (hiPSC)-derived retinal organoids. The introduction of a homozygous nonsense mutation in the *USH2A* hotspot exon-13 resulted in normal photoreceptor development but loss of ciliary localization of usherin long form B and its interacting proteins, ADGRV1 and whirlin. Notably, single-cell RNA sequencing revealed unexpected significant transcriptional changes in Müller glial cells (MGCs), suggestive of disruptions in the translation, innate immune response, and endolysosomal system. These findings suggest that, while photoreceptor cells are mildly affected by the exon-13 *USH2A* mutation, MGCs exhibit major transcriptional changes, potentially contributing to the disease progression and therefore shedding light on potential alternative therapeutic targets.

## 1. Introduction

Mutations in the human *USH2A* gene account for most cases of Usher syndrome type II (USH2), an autosomal recessive disorder characterized by progressive retinitis pigmentosa (RP) and associated hearing and balance impairment. The *USH2A* gene is one of the largest in the human genome, spanning 72 exons. Despite the diversity of variants across these exons, several mutational hotspots have been identified, with exon-13 containing two of the most common mutations found in patients [1].

According to the LOVD Database, the most frequent *USH2A* pathogenic mutation reported in almost 25% of USH2 patients is the c.2299delG, p.Glu767Serfs*21 [2,3,4], where a single base pair deletion disrupts the reading frame in the fifth laminin-type epidermal growth factor-like (EGF-Lam) domain, resulting in a premature stop codon and presumably the generation of a 786 aa truncated protein and/or nonsense-mediated mRNA decay (NMD). This variant was reported in patients from America, Africa, China, and Europe [5], reaching the highest frequency of 30.6% in Scandinavia [6] due to a homozygous *USH2A* European common ancestor [7,8]. The second most common variant is the missense mutation c.2276 G>T, p.Cys759Phe, with a frequency of 7.6%. Here, the replacement of a cysteine with a phenylalanine is thought to impair the function of usherin, the protein encoded by *USH2A*, via the disruption of a disulfide bond or the affected interactions with the extracellular matrix [9,10].

While the exact role in the retina remains unclear, usherin is thought to be involved in the regulation of protein transport between the inner and outer segments of photoreceptors cells [11]. After the first detection of usherin along the connecting cilium of murine photoreceptor cells [12], more animal models have been used, confirming the spatial localization of usherin to the periciliary membrane complex of macaque [13], zebrafish [14] and Syrian hamster [15] photoreceptors.

Murine studies have shown that usherin interacts directly with adhesion G protein-coupled receptor V1 (ADGRV1) and whirlin (WHRN) proteins through its PDZ-binding domain, forming the USH2 complex. In fact, the knock-out of whirlin long isoform in a murine model resulted in decreased usherin expression, and usherin and ADGRV1 mislocalization. Further proof of the interactions taking place in the USH2 complex, with whirlin acting as a scaffold protein, was provided by the adeno-associated viral vector (AAV)-mediated rescue of *Whrn*, which led to renewed expression of whirlin, usherin, and USH2 complex localization to the periciliary membrane complex [16]. Additionally, other proteins like harmonin (USH1C) and SANS (USH1G) interact indirectly with usherin [17,18], potentially contributing to the pathophysiology of Usher syndrome.

These interactions are critical for maintaining the structure and function of photoreceptors [19,20,21,22], and disruptions to this network are believed to play a central role in the onset of retinal degeneration seen in USH2-related conditions [16].

Despite significant advances in unraveling the genetics behind *USH2A*-related retinal degeneration, clinical treatment options remain elusive. The large size of the gene exceeds the capacity of AAV viral vectors, prohibiting the delivery of non-mutant *USH2A* form B coding sequences. Alternative delivery techniques, such as dual/triple AAV systems, intein-mediated protein splicing, or lentiviral or adenoviral vectors, attempt to overcome this problem, although limitations regarding low efficiency and harmful immune responses result in safety concerns for clinical use [23].

While the CRISPR/Cas9 technology allows the correction of pathogenic mutations [24], it has to face the poor editing efficiency in post-mitotic photoreceptor cells and the risk of off-target effects [25]. On the other hand, translational read-through drugs hold the potential of partially restoring functional protein synthesis, bypassing the premature stop codons caused by nonsense mutations, but with a limited applicability on only 20% of *USH2A* variants [26]. Similarly, an alternative therapeutic option is represented by the use of antisense oligonucleotides, e.g., QR-421a, which focus on correcting splicing defects to obtain a shortened but functional usherin [27].

Human induced pluripotent stem cells (hiPSCs) have emerged as a powerful tool for modeling human diseases in vitro [28]. In the absence of readily available patient-derived cell lines, CRISPR/Cas9 technology can be applied to generate hiPSCs carrying specific patient mutations in vitro, enabling the investigation of the pathogenic mechanisms of retinal diseases, like USH2 syndrome. Such models make it possible not only to study the disease progression, but also to develop potential gene-based therapies or drug treatments. Retinal organoids represent a rapidly advancing application of hiPSC technology. These three-dimensional in vitro structures recapitulate the layered architecture of the human retina, including rod and cone photoreceptors, and Müller glial cells, providing a more accurate representation of retinal development and disease mechanisms than traditional two-dimensional cell cultures. The differentiation of hiPSCs into retinal organoids allows the study of the impact of genetic mutations on retinal cell function in a more physiologically relevant context [29,30,31].

Importantly, *USH2A* function cannot be meaningfully investigated in undifferentiated hiPSCs, as its expression and role are restricted to specialized, mature retinal cell types. Moreover, access to patient-derived retinal tissue is limited. For these reasons, retinal organoids were established in this study as a necessary human model system.

**Scheme 1:**
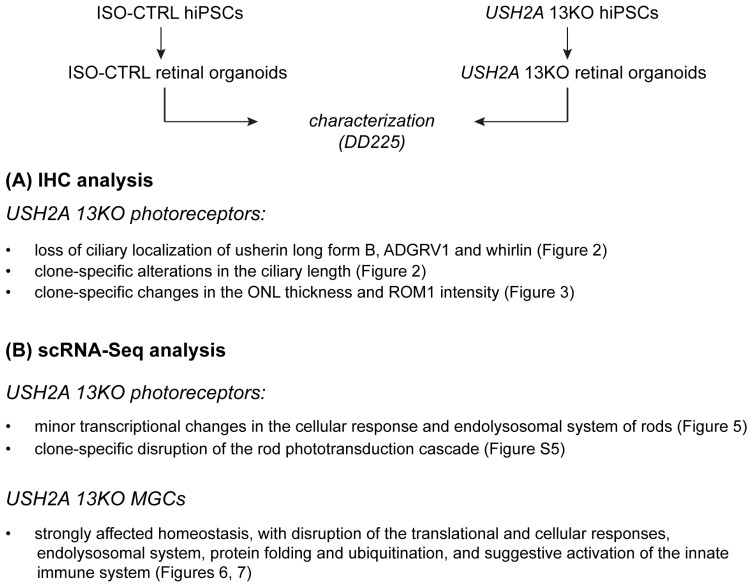
Schematics summarizing the main results obtained in this study.

Here, we describe the phenotype of differentiation day (DD) 225 retinal organoids bearing CRISPR/Cas9-engineered *USH2A* nonsense mutation in exon-13 compared with isogenic controls. Using immunohistochemical (IHC) analysis, we observed loss of the usherin protein complex at the photoreceptor cilium. In addition, single-cell RNA sequencing (scRNA-Seq) was performed with the objective of characterizing the transcriptional impact of *USH2A* loss at single-cell resolution and identifying the retinal cell types most affected by the mutation. We detected evidence for transcriptional changes suggestive of altered homeostasis and innate immune system activation in the adjacent Müller glial cells (Scheme 1).

These models offer valuable insights into the cellular and molecular mechanisms underlying the *USH2A* disease and can be used to test CRISPR/Cas9 homology-independent targeted integration therapeutic strategies [32].

## 2. Results

### 2.1. Generation and Validation of hiPSCs Carrying Homozygous Nonsense Mutations in USH2A Exon-13 by CRISPR/Cas9

LUMC0004iCTRL10 [33], hereafter referred to as “ISO-CTRL”, was used as the parental cell line to generate hiPSC subclones carrying homozygous nonsense mutations in *USH2A* exon-13 by CRISPR/SpCas9 gene editing. The knock-out (KO) strategy was designed to target the exon-13 mutational hotspot by delivering a single-stranded oligodeoxynucleotide donor, containing a TAGT nucleotide sequence to disrupt the open reading frame (Figure S1A). This *USH2A* translation stop variant results in a truncated *USH2A* open reading frame c.2553_2554insTAGT, p.(Thr852*), with a similar structure and size as predicted in other patient mutations in exon-13 (Table 1).

**Figure 1:**
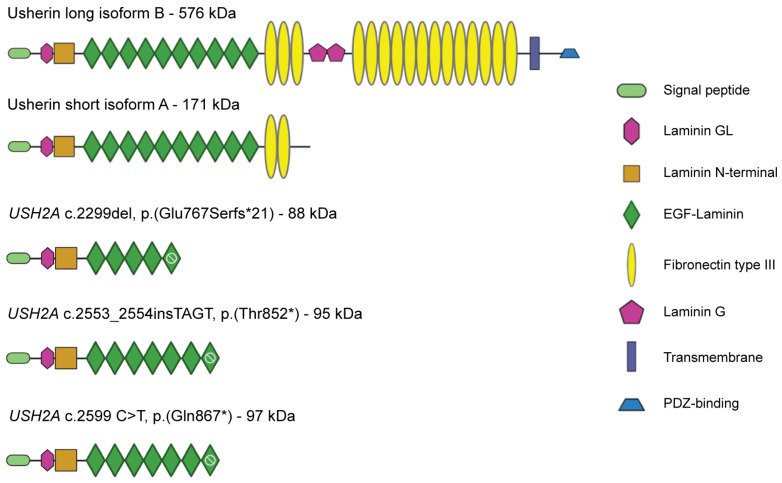
Schematics of usherin domains in the naturally occurring usherin isoforms compared to patient variants and *USH2A* 13KO. From top to bottom, depiction of the protein domains in the usherin long isoform B, the usherin short isoform A, and the usherin resulting from patient mutation c.2299del, c.2553_2554insTAGT (introduced in *USH2A* exon-13 by CRISPR/Cas9 in this study), and patient variant c.2599 C>T. Main glycosylation sites are localized in the following domains: Laminin N-terminal; EGF-Laminin numbers 2, 3, 4, 6, 7, 8, and 10; Fibronectin type III numbers 1, 2, 4, 5, 6, 7, 8, 9, 11, 12, 13, 14, 15, and 16; and Laminin G numbers 1 and 2. The molecular weight of each isoform is indicated as predicted by the ExPASy ProtParam tool. The white stop signs indicate which EGF-Laminin domain is affected upon mutation.

Two examples are the *USH2A* c.2299del, p.(Glu767Serfs*21), affecting the EGF-Lam domain number 5, and the *USH2A* c.2599 C>T, p.(Gln867*), which results in a protein containing six complete EGF-Lam domains, but of a comparable length to the c.2553_2554insTAGT, p.(Thr852*) (Figure 1).

We identified homozygous *USH2A* 13KO clones by PCR amplification of the edited locus, followed by restriction enzyme digestion with ScaI. Only hiPSC subclones displaying the fully digested amplicon were selected, demonstrating biallelic deletion and excluding the presence of a residual wild-type allele. Three independent *USH2A* 13KO subclones—labeled as 13KO E11, 13KO G8, and 13KO B7—were chosen (Table S1) and tested for the correct insertion of the stop codon through Sanger sequencing (Figure S1B, Table S2). These hiPSC lines and their derived organoids will be referred to as “13KO” throughout the manuscript. Screening for recurrent genetic abnormalities in stem cells revealed no aberrant copy number variants (Figure S1C). All three subclones showed normal karyotypes, and the short tandem repeats analysis confirmed their derivation from the same isogenic parental line (ISO-CTRL) (Figure S1D,E). When delivering SpCas9 and a targeting gRNA to hiPSCs, there is a small yet unneglectable risk that the editing will occur at undesired genomic locations [34]. For this reason, potential off-target effects were analyzed by sequencing the top 10 predicted sites and tracking of indels by decomposition (TIDE) (Figure S2, Table S2). Unfortunately, the first off-target candidate (chr6:-26266634) resides in a unique 162 bp region flanked by a stretch of 14 A-nucleotides and a stretch of 18 T-nucleotides at the 5’ and 3’ ends, respectively. Moreover, additional repetitive sequences are present both immediately upstream and downstream of the entire region of interest, and we did not succeed in cloning amplified 592 bp or 530 bp PCR fragments containing the 162 bp region. Therefore, we were not able to determine the sequence of potential off-target 1. However, the other nine sites (off-targets 2–10) were successfully sequenced and showed no indels.

### 2.2. USH2A 13KO hiPSC-Derived Retinal Organoids Show Loss of Ciliary Localization of Usherin and Its Interacting Proteins

**Figure 2:**
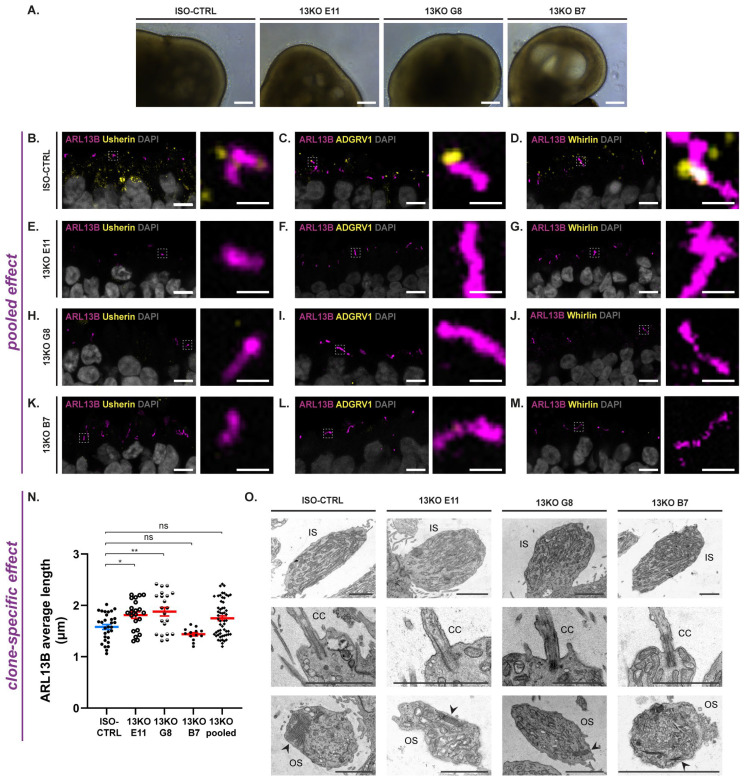
*USH2A* 13KO mutation causes loss of photoreceptor ciliary localization of USH2 proteins in retinal organoids at DD225. (**A**) Representative brightfield images of ISO-CTRL, 13KO E11, 13KO G8, and 13KO B7 retinal organoids at DD225. Scale bars: 200 µm. (**B**–**M**) Representative immunohistochemical Z-stack images of ARL13B (purple, Proteintech 17711-1-AP antibody, 1:400 dilution) and usherin ((**B**,**E**,**H**,**K**); homemade antibody, 1:2500 dilution), ADGRV1 ((**C**,**F**,**I**,**L**); homemade antibody, 1:7500 dilution), and whirlin ((**D**,**G**,**J**,**M**); Proteintech 25881-1-AP antibody, 1:400 dilution) (yellow) in ISO-CTRL and 13KO retinal organoids. Scale bars: left, 6 µm; right, 2 µm. Dashed squares highlight cilia shown at higher magnification in the adjacent images. (**N**) Quantitative analysis of ARL13B average length in ISO-CTRL and 13KO retinal organoids. Number of organoids used: ISO-CTRL *n* = 30, 13KO E11 *n* = 22, 13KO G8 *n* = 21, from four rounds of differentiation, and 13KO B7 *n* = 13, from three rounds of differentiation; 13KO pooled *n* = 56. Normality was assessed using the Shapiro–Wilk test combined with visual inspection of data distribution via QQ plots. Significance was calculated by one-way ANOVA followed by Dunnett’s multiple comparisons test and indicated as *p* < 0.05 (*), *p* < 0.01 (**), and ns (not significant). Statistical analysis: *p* = 0.037, *p* = 0.004, *p* = 0.480, and *p* = 0.067, from left to right. (**O**) Representative TEM images showing the presence of inner segment (IS)-like structures, basal body and connecting cilium (CC), and outer segment (OS)-like structures at the photoreceptor level in ISO-CTRL and 13KO retinal organoids. Arrows point to the disk membranes. Scale bars: 2 µm. Number of organoids used: ISO-CTRL *n* = 6, 13KO E11 *n* = 4, 13KO G8 *n* = 3, and 13KO B7 *n* = 3, from one differentiation. Purple side bars highlight absence of effects resulting from the KO versus presence of clone-specific effects.

Following quality assessment, the three 13KO hiPSC subclones (13KO E11, 13KO G8, and 13KO B7) were differentiated into retinal organoids along with the ISO-CTRL until DD225, using a previously published protocol [35]. Upon light microscopy, no visible differences were observed in the 13KO retinal organoids when compared to their isogenic control. At DD225, all organoids displayed typical lamination and photoreceptor segment brush borders (Figure 2A). In order to analyze more thoroughly the effects of the *USH2A* KO, immunohistochemical analyses were performed.

Due to the reported usherin long form B localization to the photoreceptor connecting cilium, we first focused on a potential phenotype concerning photoreceptor cells. When stained with a homemade antibody against the usherin isoform B [36], 13KO organoids exhibited a loss of usherin ciliary localization compared to ISO-CTRL organoids (Figure 2B,E,H,K). Previous studies in animal models showed decreased expression and mislocalization of usherin as a result of *Whrn* knock-out [16], as well as mislocalization to the photoreceptor inner segments of a truncated usherin and its interactors, ADGRV1 and whirlin, when knocking in the human c.2299delG *USH2A* variant [37]. Therefore, we were curious to investigate any potential effect of the *USH2A* 13KO on the localization of its directly interacting proteins in hiPSC-derived retinal organoids.

IHC analysis confirmed the loss of localization of ADGRV1 and whirlin at the photoreceptor cilial level (Figure 2C,D,F,G,I,J,L,M). However, when testing for the presence of whirlin on a Western Blot, the protein was detected at similar levels in both the ISO-CTRL and in the 13KO retinal organoid lysates (Figure S3A–C), suggesting redistribution of whirlin throughout the photoreceptor cells.

We used ADP-ribosylation factor-like protein 13B (ARL13B) as a marker for the photoreceptor cilium axoneme, and we observed a statistically significant increase in the average ciliary length in two out of three 13KO subclones when compared to the ISO-CTRL, but not in the 13KO pool (Figure 2N).

The development of intact photoreceptor ciliary structures in the 13KO retinal organoids was further confirmed by transmission electron microscopy (TEM) analysis. The organoids differentiated from both the 13KO and the parental hiPSC lines showed inner segment (IS)-like structures, characterized by the presence of mitochondria, a connecting cilium (CC), where usherin localizes in retinal organoids, with a clear basal body, and outer segment (OS)-like structures, with photoreceptor disks at varying stages of maturation. Some 13KO organoids, like 13KO subclone B7, displayed poorly organized disks, while others exhibited a more structured arrangement (Figure 2O).

**Figure 3:**
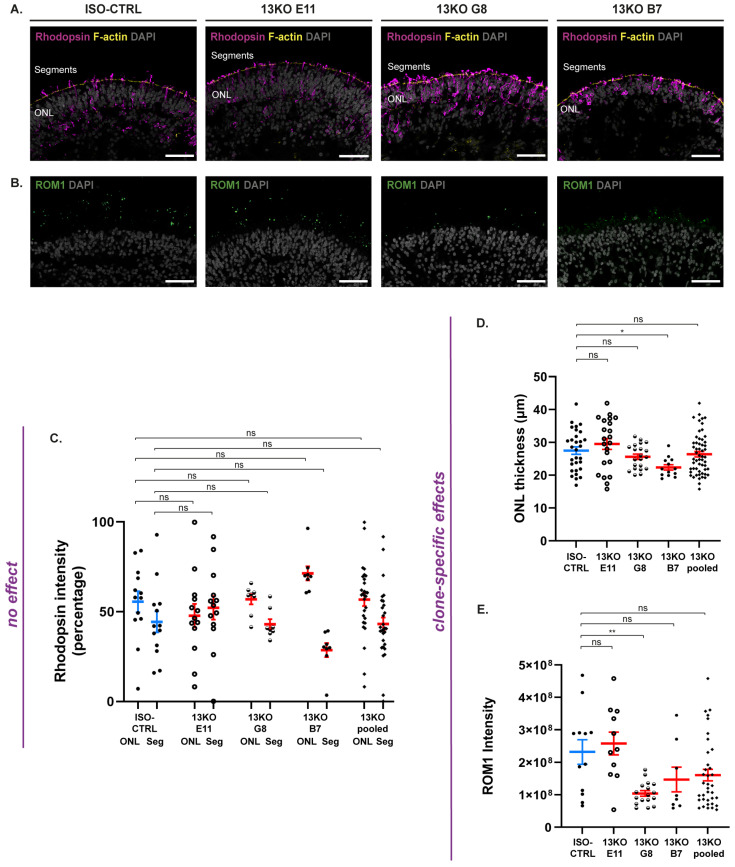
Phenotypic analysis of the photoreceptor layer in retinal organoids upon *USH2A* loss at DD225. (**A**) Representative immunohistochemical images of rhodopsin (purple, Millipore MAB5356 antibody, 1:500 dilution) and F-actin (yellow, Thermo Fisher Scientific r-415, 1:300 dilution) in ISO-CTRL, 13KO E11, 13KO G8, and 13KO B7 retinal organoids at DD225. (**B**) Representative immunohistochemical images of ROM1 (green, Proteintech 21984-1-AP antibody, 1:400 dilution) in ISO-CTRL, 13KO E11, 13KO G8, and 13KO B7 retinal organoids at DD225. (**A**,**B**) Scale bars: 50 µm. (**C**) Quantitative analysis of rhodopsin intensity distribution between the outer nuclear layer (ONL) and the photoreceptor segments (Seg) in ISO-CTRL and 13KO retinal organoids. Number of organoids used: ISO-CTRL *n* = 13, 13KO E11 *n* = 13, 13KO G8 *n* = 8, and 13KO B7 *n* = 8; 13KO pooled *n* = 29, from four rounds of differentiation. Statistical analysis: *p* = 0.688, *p* = 0.999, *p* = 0.212, and *p* = 0.999, from left to right. (**D**) Quantitative analysis of the ONL thickness in ISO-CTRL and 13KO retinal organoids. Number of organoids used: ISO-CTRL *n* = 30, 13KO E11 *n* = 22, and 13KO G8 *n* = 21, from four rounds of differentiation, and 13KO B7 *n* = 13, from three rounds of differentiation; 13KO pooled *n* = 56. Normality was assessed using the Shapiro–Wilk test combined with visual inspection of data distribution via QQ plots. Significance was calculated by one-way ANOVA followed by Dunnett’s multiple comparisons test. Statistical analysis: *p* = 0.584, *p* = 0.650, *p* = 0.037, and *p* = 0.838, from left to right. (**E**) Quantitative analysis of ROM1 intensity in ISO-CTRL and 13KO retinal organoids. Number of organoids used: ISO-CTRL *n* = 12, 13KO E11 *n* = 11, and 13KO G8 *n* = 17, from four rounds of differentiation, and 13KO B7 *n* = 8, from three rounds of differentiation; 13KO pooled *n* = 36. Normality was assessed using the Shapiro–Wilk test combined with visual inspection of data distribution via QQ plots. Significance was calculated by one-way ANOVA followed by Dunnett’s multiple comparisons test. Statistical analysis: *p* = 0.920, *p* = 0.0005, *p* = 0.207, and *p* = 0.120, from left to right. Purple side bars highlight absence of effects resulting from the KO versus presence of clone-specific effects. (**C**–**E**) Normality was assessed using the Shapiro–Wilk test. Data distribution was visually inspected using QQ plots. Significance was calculated by one-way ANOVA followed by Dunnett’s multiple comparisons test and indicated as *p* < 0.05 (*), *p* < 0.01 (**), and ns (not significant). When data violated normality assumptions, group differences were assessed using the Kruskal–Wallis test followed by Dunn’s multiple comparisons test.

Some retinal diseases in mice are associated with disrupted trafficking of rhodopsin to the photoreceptor outer segments, and its subsequent retention in the cell bodies. In this study, we quantified the distribution of rhodopsin between the outer nuclear layer (ONL) and the photoreceptor segments (Figure 3A), and we observed no differences in the 13KO when compared to the parental line retinal organoids (Figure 3C).

Furthermore, we investigated whether the *USH2A* 13KO caused an alteration in the volume of the photoreceptor layer. The outer nuclear layer thickness was found to be not statistically significantly different in the 13KO compared to the control, except for the differentiations of subclone B7, which displayed a statistically significant decrease (Figure 3D). An effect was observed at the photoreceptor disk level of differentiations of the 13KO subclone G8 retinal organoids, which showed a statistically significant lower intensity of the outer segment disk marker rod outer segment membrane protein 1 (ROM1), yet this was not detected in the analysis of the 13KO pool (Figure 3B,E).

### 2.3. scRNA-Seq Analysis Reveals Transcriptional Changes Suggestive of Altered Homeostasis and Innate Immune System Activation in the USH2A 13KO Müller Glial Cells

**Figure 4:**
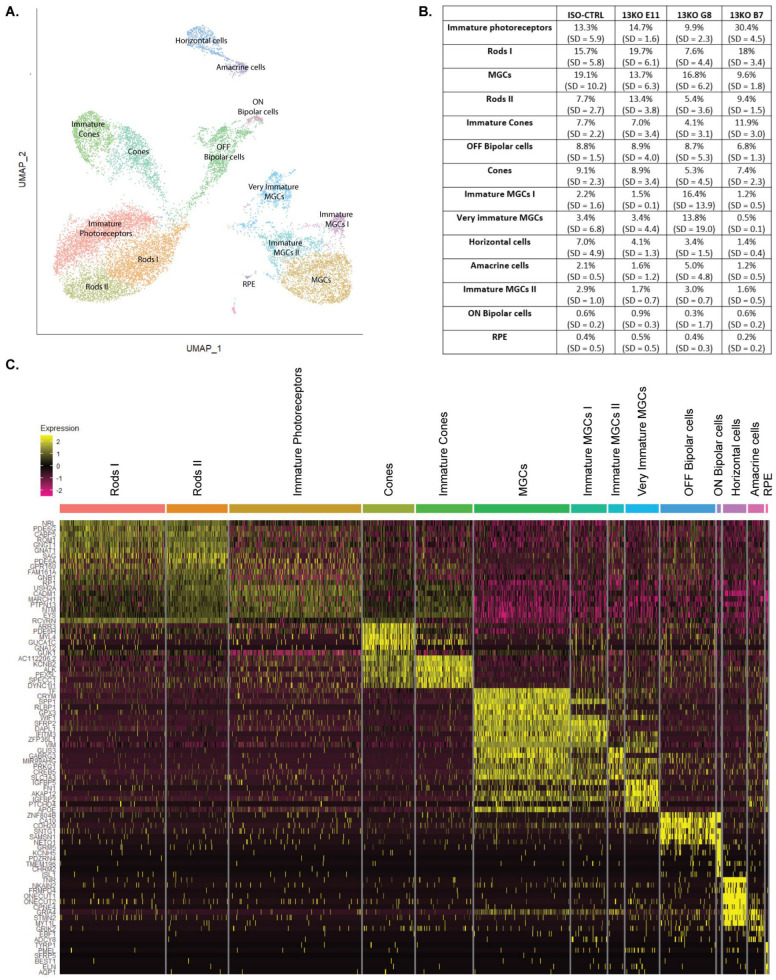
scRNA-Seq analysis confirms the presence of all major retinal cell types in hiPSC-derived *USH2A* 13KO and isogenic control retinal organoids. (**A**) UMAP displaying 14 identified distinct cell clusters. (**B**) Table showing the percentage of each cell type across conditions. Values are expressed as mean ± SD (standard deviation). A total of 14 individual organoids from one differentiation were used in 14 scRNA-Seq and distributed as follows: ISO-CTRL *n* = 4, 13KO E11 *n* = 4, 13KO G8 *n* = 3, and 13KO B7 *n* = 3. (**C**) Heatmap of top markers assigning cell cluster identities.

Next, we used single-cell RNA sequencing (scRNA-Seq) to analyze the transcriptomic differences between the 13KO and ISO-CTRL organoids. Fourteen distinct clusters were identified based on their gene expression profiles, with all major retinal cell types, including photoreceptor cells (rods and cones), Müller glial cells (MGCs), ON and OFF bipolar cells, amacrine cells, and horizontal cells (Figure 4A).

Some clusters corresponded to more immature stages of development, specifically of photoreceptor cells (labeled as “Immature photoreceptors” and “Immature Cones”), and MGCs (including “Immature MGCs I”, “Immature MGCs II”, and “Very Immature MGCs”). Two rod photoreceptor cell subtypes, “Rods I” and “Rods II”, were identified. Upon further analysis, Rods I and Rods II seemed to have a highly similar profile, thus we decided to merge them into a single “Rods” cluster for downstream differential gene expression and pathway enrichment analyses.

A high degree of variability was observed in the cell-type composition of the retinal organoids across conditions. Despite immature photoreceptors and MGCs clusters being more prevalent in some organoids (Figure 4B), none of the processed samples could be considered outliers, and they were all included in the downstream analysis. The enrichment in expression of a variable number of key cell-type specific genes displayed on a heatmap helped in assigning the cluster identities (Figure 4C). Specifically, we obtained a list of markers enriched in each subset of cells through the FindAllMarkers function in R software package Seurat (v4.4.0) for statistical computing and checked their cell-specific expression [38,39,40]. For instance, *NRL* identified the rod photoreceptor cells, *ARR3* was exclusively expressed in the cones, *TF* transcript levels were found enriched in the MGCs, and *ONECUT1* was used to classify the horizontal cells.

We performed a differential gene expression analysis to investigate any potential changes between the 13KO subclones and the isogenic control organoids. The expression of *USH2A*, *ADGRV1* and *WHRN* was predominantly observed in photoreceptor cells and MGCs (Figure S4A,E,I).

Interestingly, the expression levels of *USH2A* (Figure S4B–D), *ADGRV1* (Figure S4F–H), and *WHRN* (Figure S4J–L) were not statistically significantly different in the rods, cones, and MGCs of the 13KO lines when compared to the ISO-CTRL. No significant differential expression was found in some retinal cell types, such as amacrine cells or ON bipolar cells. Surprisingly, only three differentially expressed genes (DEGs) were detected in the cones of the 13KO subclone B7 (*ASIC2*, *CC2D2A*, and *ARHGAP35*), whereas *PSKH2* was differentially expressed exclusively in the subclone E11. Of these, *CC2D2A* was the only gene directly related to the cilia formation. However, the most substantial differences in gene expression were observed in the rods and MGC clusters.

**Figure 5:**
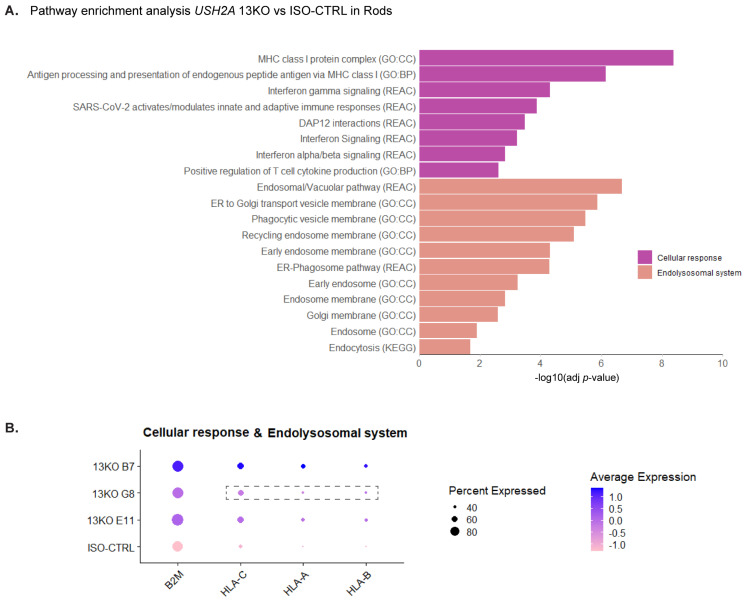
scRNA-Seq analysis of DD225 *USH2A* 13KO retinal organoids shows disruptions in the endolysosomal system and cellular response in rod photoreceptors compared to isogenic controls. (**A**) Pathway enrichment analysis of differentially expressed genes (DEGs) in rods, with similar terms grouped into categories. (**B**) Statistically significant DEGs in at least two *USH2A* 13KO subclones in rods. Dashed boxes highlight genes that did not exhibit differential expression in a specific subclone compared to ISO-CTRL. There are no significantly downregulated DEGs. Number of organoids used: ISO-CTRL *n* = 4, 13KO E11 *n* = 4, 13KO G8 *n* = 3, and 13KO B7 *n* = 3 from the same differentiation round. Differential expression at the single-cell level was assessed using Wilcoxon rank-sum tests as implemented in Seurat, with *p*-values adjusted for multiple testing using the Benjamini–Hochberg false-discovery rate (FDR). Genes with FDR < 0.05 were considered differentially expressed.

The DEGs detected in the rod photoreceptor cells were subjected to pathway enrichment analysis (based on Gene Ontology, KEGG and Reactome), which identified two main categories of affected biological processes. We labeled them as “Cellular response” and “Endolysosomal system”. The first group included terms involved in the activation of the interferon signaling and antigen presentation, whereas the second category included terms related to the endosome membrane and vacuolar pathway (Figure 5A).

To gain further insight into the specific genes involved, dot plots were used to visualize the differential expression of genes within each pathway category. Interestingly, the terms related to “Cellular response” and “Endolysosomal system” in the rods were associated with the same set of DEGs, namely *B2M*, *HLA-C*, *HLA-A*, and *HLA-B* (Figure 5B).

Interestingly, only the *USH2A* 13KO subclone B7 retinal organoids showed a significant decrease in the photoreceptor ONL thickness (Figure 3D). On scRNA-Seq, only the rod photoreceptors in the three *USH2A* 13KO subclone B7 retinal organoids showed statistically significantly downregulated DEGs related to the phototransduction cascade, such as *PDE6A*, *PDE6G*, *GNAT1* and *SAG* (Figure S5). The decrease in ONL thickness and the presence of DEGs related to phototransduction were detected exclusively in 13KO B7, but not in 13KO E11 or 13KO G8 retinal organoids. It is therefore not clear whether this phenotype is related to the *USH2A* mutation.

**Figure 6:**
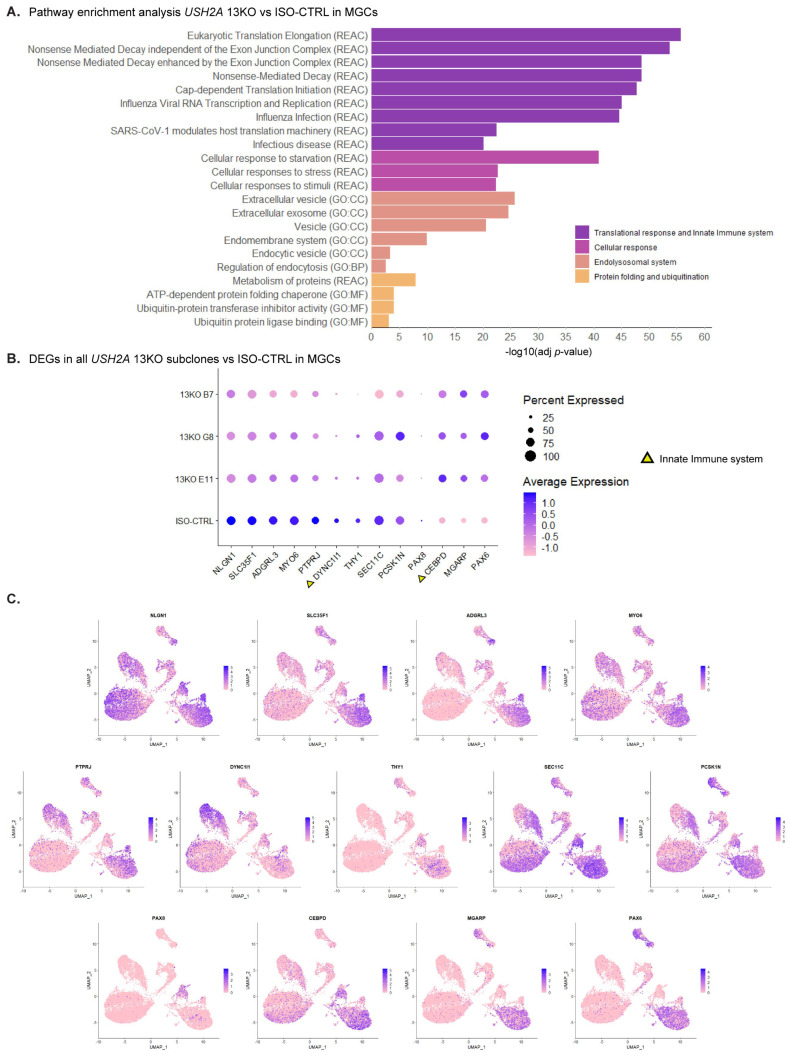
scRNA-Seq analysis of DD225 *USH2A* 13KO retinal organoids shows major disruptions in the homeostasis of Müller glial cells compared to isogenic controls. (**A**) Pathway enrichment analysis of differentially expressed genes (DEGs) in MGCs, with similar terms grouped into categories. (**B**) Dot plot of the statistically significant DEGs found in the MGCs of all three *USH2A* 13KO subclones. Triangles indicate the genes involved in pathways related to the innate immune system. (**C**) UMAPs of the 13 common DEGs detected. Number of organoids used: ISO-CTRL *n* = 4, 13KO E11 *n* = 4, 13KO G8 *n* = 3, and 13KO B7 *n* = 3 from the same differentiation round. Differential expression at the single-cell level was assessed using Wilcoxon rank-sum tests as implemented in Seurat, with *p*-values adjusted for multiple testing using the Benjamini–Hochberg false-discovery rate (FDR). Genes with FDR < 0.05 were considered differentially expressed.

A similar pathway enrichment analysis was performed on the MGCs. Unexpectedly, a major and more complex phenotype was observed in this cluster, with the identification of four distinct groups of pathway terms. The most relevant pathway terms per category occurring in at least two subclones were selected and displayed with the lowest detected adjusted *p*-value in a bar plot (Figure 6A).

The first one was a mixed group of terms related to “Translational response and Innate Immune system”. This included terms referring to translation initiation and elongation, nonsense mediated decay and viral infection responses triggering the activation of the innate immune system. The second category grouped together those terms related to other “Cellular response”, spanning from response to stress to response to stimuli. The third group was labeled as “Endolysosomal system”, including terms about vesicles and the endocytosis process, similarly to what was observed in the rod photoreceptor cells. The fourth category was named “Protein folding and ubiquitination”, pointing to disturbances in protein quality control. Surprisingly, terms in the first three categories reached highly significant adjusted *p*-values, with peaks of 10^−55^, indicating an unexpected major phenotype in the MGCs caused by the *USH2A* 13KO mutation.

When looking into the genes associated with the terms indicative of a disrupted homeostasis, we identified thirteen genes being differentially expressed in the MGCs of all three 13KO subclones, compared to the ISO-CTRL (Figure 6B). Amongst these, *CEBPD* encodes for a transcription factor regulating the expression of genes involved in the immune and inflammatory responses, *DYNC1I1* is involved in the motility of vesicles and organelles though microtubules, and *MYO6* participates in the vesicular membrane trafficking and clathrin-mediated endocytosis.

The cluster enrichment of the transcripts related to these common 13 DEGs in the MGCs was depicted via UMAP plots (Figure 6C). This representation method was quite informative, indicating how some of the genes had a preferential expression in the MGC cluster, like *THY1*, whereas others were more ubiquitously expressed, like *SEC11C*.

**Figure 7:**
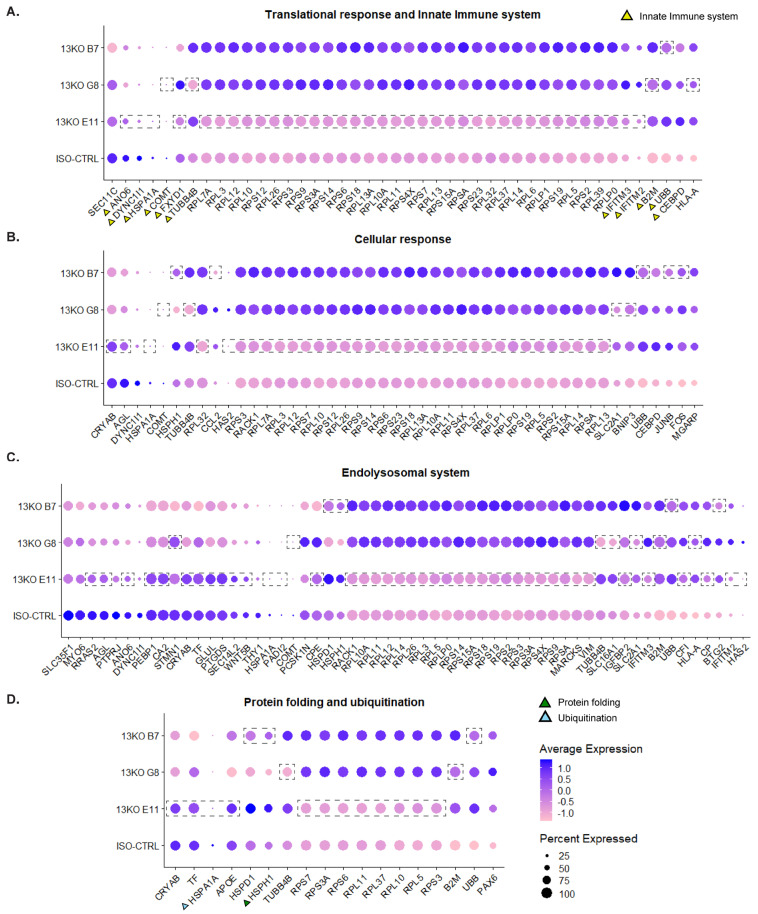
Differential expression analysis of Müller glial cells compared to isogenic controls. (**A**–**D**) Statistically significant DEGs in at least two *USH2A* 13KO subclones involved in translational response and innate immune system (**A**), cellular response (**B**), endolysosomal system (**C**), and protein folding and ubiquitination (**D**). Dashed boxes highlight genes that did not exhibit differential expression in a specific subclone compared to ISO-CTRL. Triangles indicate those genes that are exclusively involved in a single pathway within the macro-category. Number of organoids used: ISO-CTRL *n* = 4, 13KO E11 *n* = 4, 13KO G8 *n* = 3, and 13KO B7 *n* = 3 from the same differentiation round. Differential expression at the single-cell level was assessed using Wilcoxon rank-sum tests as implemented in Seurat, with *p*-values adjusted for multiple testing using the Benjamini–Hochberg false-discovery rate (FDR). Genes with FDR < 0.05 were considered differentially expressed.

We decided to then expand our analysis, including also those genes that were differentially expressed in at least two out of three subclones and visualize them in separate dot plots organized per category (Figure 7).

The two MGC-associated DEGs that most caught our attention were *IFITM2* (interferon-induced transmembrane protein 2) and *IFITM3* (interferon-induced transmembrane protein 3), which turned out to be associated with terms related to both “Innate Immune system” and “Endolysosomal system” (Figure 7A,C). *IFITM2* and *IFITM3* are known to be involved in the anti-viral response mechanisms [41,42,43], suggesting the transcriptional activation of the innate immune system upon the *USH2A* 13KO. When visualized on a UMAP, *IFITM2* and *IFITM3* turned out to be expressed almost exclusively in the MGCs (Figure S7A,B). Several other genes involved in the innate immune system were differentially expressed in the Müller glial cell cluster, i.e., *ANO6*, *DYNC1I1*, *HSPA1A*, *COMT*, *FXYD1*, *TUBB4B*, *B2M*, *UBB*, and *CEBPD* (Figure 7A). 

Knowing that *IFITM2* and *IFITM3* are interferon-stimulated genes, we wondered if there were any interferon-expressing cells in our retinal organoids. Therefore, we checked the levels of *IFNA1* (Interferon Alpha-1), *IFNA2* (Interferon Alpha-2), *IFNB1* (Interferon Beta-1), and *IFNG* (Interferon Gamma) in our scRNA-Seq dataset, but we found no detectable expression of these genes in the ISO-CTRL or 13KO retinal organoids.

Volcano plots were used to compare the results of the differential gene expression analysis performed in rods (Figure S6A) and MGCs (Figure S6B) by displaying simultaneously the magnitude of the expression changes (log2 fold change) and their statistical significance according to the adjusted *p*-value. This representation provides an overview of the genes that are up- or downregulated in the mutants compared to the ISO-CTRL, allowing us to better visualize effect sizes. The plots confirm that, while expression changes are relatively modest in the rods, the mutant MGCs exhibit larger-magnitude transcriptional changes, with many more highly significant DEGs.

### 2.4. Expression of USH2A Transcripts Encoding Usherin Isoform A and Usherin Isoform B in the Retinal Organoids

Once we assessed the genotypic impact of the *USH2A* 13KO on the retinal cell types, and specifically on the Müller glial cells, we investigated whether this was possibly due to the expression of a truncated mutant usherin in the photoreceptor cells or in the MGCs. To accurately distinguish the *USH2A* isoforms, the reference genome annotation was customized. All *USH2A* transcripts were removed, and only the transcripts encoding for the short isoform A and the long isoform B were reintroduced in our scRNA-Seq dataset, annotated as two independent gene features, namely “*USH2A*-short” and “*USH2A*-long”. This enabled us to perform an isoform-specific expression analysis in the rods (Figure S8A,D), cones (Figure S8B,E), and MGC clusters (Figure S8C,F).

The violin plots showed that the *USH2A*-short transcript was expressed at a much lower level than the *USH2A*-long transcript across the analyzed clusters. Nonetheless, the *USH2A*-short transcript appeared to be enriched in the rod and cone photoreceptor cells, whereas its expression in the MGCs was almost negligible. These findings suggest that the genotypic effect observed on the MGCs’ homeostasis is likely driven by events or signals originating from the photoreceptor cells, where the short form A is mainly expressed.

### 2.5. The USH2A 13KO Müller Glial Cells Do Not Exhibit Morphological Changes

We proceeded to analyze any potential resulting phenotypic changes in the Müller glial cells. For instance, the presence of reactome terms like “response to stress” led us to expect high presence of glial fibrillary acidic protein (*GFAP*) transcript and protein in the MGCs [44,45]. Surprisingly, we observed that only a few cells in the MGC cluster showed presence of *GFAP* transcript, and the level of expression was relatively low (Figure S7C). The Western Blot and immunohistochemical analyses revealed no statistically significant differences in GFAP intensity in the 13KO organoids compared to the isogenic control line (Figures S3D–F and S7D,F). Similarly, the staining against CD44, a marker for the apical processes of MGCs, revealed no significant changes (Figure S7E,G).

Altogether, these data show an unforeseen major genotypic effect on the MGCs caused by the *USH2A* 13KO mutation, which does not translate into morphological changes.

## 3. Discussion

In this study, we successfully generated by CRISPR/SpCas9 technology three hiPSC subclones carrying a homozygous nonsense mutation in *USH2A* exon-13 and differentiated them into retinal organoids for 225 days. Unfortunately, the most probable off-target (chr6:-26266634) was not analyzed, due to unsuccessful direct Sanger sequencing of 592 bp and 530 bp PCR fragments, as well as failed cloning of DNA amplicons containing the 162 bp region encompassing the potential off-target site 1 from the ISO-CTRL genome and from the genomes of *USH2A* 13KO clones B7, E11, or G8. Interestingly, this 162 bp DNA region is unique for the human genome. It is not evolutionary conserved, and it is not present in the genome of non-human primates. Furthermore, it is not detected in mRNA or other RNA transcripts. Notably, only a single 89301 bp bacterial artificial chromosome (BAC) containing this 162 bp sequence has been reported in the NCBI human genome GenBank database. The single report of this sequence might reflect technical difficulties in sequencing the human genome at or near this 162 bp region (GenBank: AL031777.5). Nevertheless, we obtained PCR fragments of the expected sizes, including the adjacent A- and T-nucleotide stretches, suggesting that the DNA region is part of the human genome but difficult to clone and sequence. Given that off-target site 1 represents the most probable off-target, we considered whether potential gene editing at this locus could have affected our results on the loss of usherin long form B at the 13KO photoreceptor cilium or other phenotypes or DEGs observed in 13KO B7, 13KO E11, or 13KO G8 retinal organoids. In the case of a heterozygous indel, or compound heterozygous indels affecting both alleles, it is unlikely that these variants influenced the results obtained, as the putative indels do not affect exonic regions of transcribed genes [46]. 

The overall morphology of *USH2A* 13KO photoreceptor structures appeared largely unaffected. The distribution of rhodopsin, a key rod photoreceptor protein, between the outer nuclear layer (ONL) and the photoreceptor segments was preserved in all mutant subclones. However, only *USH2A* 13KO subclone B7 showed a significant decrease in ONL thickness, while 13KO G8 exhibited a difference in the intensity of the disk marker ROM1 when compared to the controls. Photoreceptor disks showed varying stages of outer segment development in the TEM images.

Despite the average ciliary length being significantly increased in two 13KO subclones, this did not reach statistical significance in the comparison of the 13KO pool to the ISO-CTRL. Our data do not fully support the affected ciliogenesis detected in cells derived from non-syndromic RP and USH patients, where the fibroblasts carrying mutations in the *USH2A* gene developed a 1.5-fold longer cilium than in the controls [31]. However, the findings related to the ciliary length in our 13KO retinal organoids are in contrast with a previous investigation in mice, where the *Ush2a^delG/delG^* variant caused the early-onset shortening of the photoreceptor cilium [37].

Variation across differentiation batches of retinal organoid subclones was observed in some of the traits analyzed by immunohistochemistry. The clone-specific effects on ARL13B length, ONL thickness, and ROM1 intensity upon the KO might be explained by the inherent stochasticity of retinal organoid differentiation. In fact, subtle differences in the efficiency of cell type specification or maturation between multiple rounds of differentiation can result in variability in marker expression or structural features.

This study also observed that the long isoform B of usherin, along with its interacting proteins ADGRV1 and whirlin, were no longer detectable at the photoreceptor ciliary level in the *USH2A* 13KO retinal organoids, compared to the isogenic controls. Despite the biological variability, this phenotype was confirmed across independent batches, supporting the reproducibility and robustness of our findings. Yet the presence of their transcripts in our scRNA-Seq data, as well as the detection of whirlin via Western Blot, suggest that these proteins can still be produced but might be mislocalized in the photoreceptors or trapped in the endoplasmic reticulum (ER) or Golgi apparatus. This is consistent with what was previously observed in a knock-in mouse expressing the common human disease mutation c.2299delG. In that model, usherin was truncated and glycosylated, and accumulated in the ER, causing cellular stress and disrupting the localization of ADGRV1 and whirlin at the periciliary membrane complex. As a result, they were trapped in vesicles in the inner segments [37].

The co-dependence of proteins within the USH2 complex had been already highlighted earlier in a knock-out murine model of *Whrn*, where usherin expression was decreased and its localization in the ciliary pocket compromised [16].

The impaired assembly and function of the usherin interactome in response to *USH2A* mutations was further investigated in hiPSC-derived retinal organoids. Whilst usherin and ADGRV1 were correctly detectable at the base of the connecting cilium in the isogenic controls, both proteins were mislocalized to the inner segments of organoids carrying *USH2A* variants responsible for either retinitis pigmentosa or USH2 [31].

The usherin antibody used in this analysis was raised against the C-terminal region of the protein and therefore only able to detect the usherin long isoform B. Antibodies that specifically detect the short form of usherin, or the C-terminally truncated 13KO short form, are not available. Our data demonstrate that the mutant 13KO *USH2A* transcripts, which encompass the 3′ untranslated region of the 13KO long and short splice forms of *USH2A*, do not undergo statistically significant nonsense-mediated mRNA decay (NMD). The NMD-related reactome terms in the MGCs suggest that the NMD might be affected to an extent that is no longer sufficient to clear the *USH2A* transcripts containing premature stop codons. The absence of NMD suggests that the 13KO mRNA transcripts are still available to produce the 13KO short form of usherin.

Further studies are required to investigate whether the 13KO short form usherin in photoreceptors is produced and stable, and whether the 13KO short form usherin or another protein or signal from the photoreceptor cells is responsible for evoking the loss of homeostasis in adjacent Müller glial cells.

Our hypothesis is that a usherin mutant short form A (851 aa) or another protein might be secreted by the photoreceptor cells and endocytosed by the MGCs, triggering a cellular innate immune response, due to being recognized as a “foreign” or misfolded protein. This could explain the significant dysregulation of the endolysosomal system and the presence of gene ontology terms indicative of protein misfolding, leading to the ubiquitination process. The entire cellular homeostasis in the MGCs of 13KO retinal organoids was compromised, spanning from translation to cellular response to stimuli. While the transcriptional signature of *USH2A* 13KO MGCs reveals enrichment of immune-related pathways, functional validation of innate immune system activation was beyond the scope of the current study. Future investigation will assess cytokine secretion (i.e., IL-6, TNF-α, and CXCL10) and activation of interferon signaling, as measured by STAT1 phosphorylation. Additional validation could be obtained by exposing the organoids to innate immune stimuli, such as lipopolysaccharide (LPS) or cyclic GMP-AMP (cGAMP), to determine whether *USH2A* alters cellular responsiveness to inflammatory or interferon-mediated challenges.

The detection of the *USH2A* transcript encoding the short usherin isoform A primarily in rods, and to a lesser extent in cones, rather than in the Müller glial cells, suggests that the MGCs function as sensors of events occurring in the photoreceptor cells and respond by exhibiting signs of disrupted homeostasis. Differential expression of interferon-stimulated genes (*IFITM2* and *IFITM3*) was observed, indicating an innate immune response, similarly observed upon viral infection. In fact, the release of type I and type II interferons induced by a pathogenic challenge upregulate *IFITM3* expression, which inhibits the entry of viruses into the host cells. Localizing to the endosomal compartment, *IFITM3* alters the rigidity and pH of cellular membranes, preventing the virus replication [42,47]. In this study, no expression of the interferon ligands *IFNA1*, *IFNA2*, *IFNB1*, or *IFNG* was observed. It is possible that the IFNs or cytokines are produced in the organoids, but their transcript levels fall below the detection limit of scRNA-Seq. Additionally, *IFITM2* and *IFITM3* might also be induced through interferon-independent mechanisms, including cellular stress pathways or pattern-recognition receptor signaling [47,48].

Further studies are required to elucidate the molecular mechanisms underlying the involvement of a cellular innate immune response in the *USH2A* 13KO retinal organoids. In follow-up experiments, we plan to transduce retinal organoids with AAVs expressing HA-tagged *USH2A* 13KO cDNA to determine whether a short mutant usherin isoform A is produced, to define its subcellular localization, and to assess whether and when uptake by the MGCs occurs. Pulse–chase assays, co-culture experiments, or tagged usherin secretion/uptake studies could also contribute to gain more insights on this topic.

Interestingly, the MGC genotypic changes detected in our scRNA-Seq dataset were not reflected in a pronounced altered morphological phenotype. Despite the relevance of reactome terms indicating the activation of the MGC response to stress, the intensity of GFAP, a marker of gliosis, was not statistically different in the 13KO subclones compared to the ISO-CTRL. Additionally, the apical villi of MGCs, as observed through CD44 staining and by TEM, were preserved in the mutant organoids, suggesting that there were no significant structural defects in the MGCs upon the KO of *USH2A* in exon-13.

This analysis provided a comprehensive overview of the molecular pathways most affected by the *USH2A* 13KO mutation. Notably, genes related to the phototransduction cascade, such as *PDE6A*, *PDE6G*, *GNAT1* and *SAG*, were found significantly downregulated, but exclusively in the rods of *USH2A* 13KO subclone B7. Although the photoreceptor cells showed some alterations in their expression profile, the most pronounced genetic changes occurred in the MGCs. Compared to rods, MGCs exhibited both larger effect sizes and stronger statistical significance in response to the KO, indicating that MGCs are more transcriptionally affected by the genetic perturbation. In fact, unexpectedly, these cells showed transcriptional evidence of disruptions in several critical processes, including translation, immune response, and protein folding. The involvement of MGCs in Usher 1C disease has been previously investigated [49,50,51], but the exact mechanisms of action remain unclear.

However, while the phenotypic characterization of the *USH2A* 13KO organoids by immunohistochemistry was performed across at least three independent differentiation rounds, the 14 scRNA-Seq analyses were conducted on 14 individual retinal organoids (ISO-CTRL *n* = 4, 13KO E11 *n* = 4, 13KO G8 *n* = 3, and 13KO B7 *n* = 3) derived from a single differentiation batch. As such, the findings should be interpreted with caution, as batch-specific effects cannot be excluded. Future studies will aim to validate the reproducibility and robustness of the observed transcriptional signatures. The short 851 aa usherin resulting from our KO design in *USH2A* exon-13 mimics the truncated usherin produced in several patients with mutations in exon-13, such as c.2299delG and c.2599 C>T (Table 1). Since those proteins share the same size range and functional domains, we can hypothesize that the phenotype observed in our 13KO retinal organoids faithfully recapitulate the phenotype of patients carrying other mutations in *USH2A* exon-13, shedding light on a better comprehension of the pathogenic mechanisms behind Usher syndrome type II.

In conclusion, this study suggests that, while the photoreceptor cells in the retinal organoids showed a loss of shuttling of usherin complex proteins to the cilium, the rod and cone photoreceptors were not severely affected at the gene expression level by the introduction of a nonsense mutation in exon-13 of the *USH2A* gene, whereas the Müller glial cells were strongly impacted, possibly playing a significant role in the pathogenesis of *USH2A*-related retinal diseases. Further investigation is needed to better unravel the mechanisms underlying the activation of the innate immune system and its implications for retinal disease progression and retinal gene therapy.

## 4. Materials and Methods

### 4.1. CRISPR/Cas9-Mediated Genome Editing in hiPSCs

The SpCas9-gRNA targeting *USH2A* exon-13 (crRNA) was selected based on the predicted highest efficiency and lowest number of off-target sites according to the integrated DNA technology (IDT) software (https://eu.idtdna.com/, access date 1 August 2022). A single-stranded oligodeoxynucleotide was designed to contain 4 extra nucleotides forming a premature stop codon and disrupting the reading frame after integration at the desired locus (Table S2). The correct insertion in the genome recreates the ScaI restriction site, useful for screening purposes. Both the crRNA and the ssODN (IDT, Madison, WI, USA) were dissolved in the Nuclease-Free Duplex Buffer (IDT, Madison, WI, USA) to 100 µM stock. The ssODN was further resuspended in Resuspension Buffer R (IDT, Madison, WI, USA) to working concentration. The crRNA and the tracrRNA were mixed together and incubated at 95 °C for 5 min, in the presence of the Nuclease-Free Duplex Buffer to obtain the gRNA duplex. Once cooled down to room temperature (RT), the gRNA was incubated for 20 min with SpCas9 Nuclease V3 (IDT, Madison, WI, USA) in Resuspension Buffer R, to form the ribonucleoprotein (RNP) complex. hiPSCs were seeded on Matrigel-coated 6-well plates (Corning, Glendale, AZ, USA) and passaged mechanically with Gentle Cell Dissociation reagent (GCDR) (STEMCELL Technologies, Cologne, Germany), according to manufacturer’s instructions. When ready for genome editing, the culturing medium was substituted with 1 mL/per well of Accumax (STEMCELL Technologies, Cologne, Germany), to improve single-cell dissociation. Cells were incubated for 7 min at 37 °C and collected in DMEM/F12 (ThermoFisher Scientific, Waltham, MA, USA). An aliquot of 3.6 × 10^5^ cells was centrifuged at 1100 rpm for 3 min and resuspended in 27 µL of buffer R. The delivery of 4 µL of the ssODN + 2 µL of the RNP complex to 18 µL of cell suspension was mediated by electroporation, using the Neon Transfection System (ThermoFisher Scientific, Waltham, MA, USA) with pulse conditions set to 1200 V/30 ms/1 pulse. Thereafter, the sample was immediately transferred into a new 12-well plate containing prewarmed medium. Cell handling after electroporation and the colony screening technique are described in the Supplementary Methods. Materials can be found in Table S3.

### 4.2. Cell Culture and Retinal Organoid Differentiation

We previously described the male control line LUMC0004iCTRL10 [33], here labeled as ISO-CTRL and used as a parental cell line. Characterization of undifferentiated state and pluripotency is available under the hPSC registry name LUMCi029-B. Cryopreserved hiPSCs (passage number p3) were thawed at 37 °C, resuspended in mTeSR plus (STEMCELL Technologies, Cologne, Germany) + 0.1% Fasudil HCl (Focus Biomolecules, Plymouth Meeting, PA, USA), and seeded on Matrigel-coated 6-well plates. After three weeks (passage number p9), confluent hiPSCs were resuspended in mTeSR plus + 10 µM blebbistatin (Abcam, Waltham, MA, USA). One million cells were seeded in each agarose micro-mold (Merck, Schiphol-Rijk, The Netherlands) placed in a 12-well plate and incubated overnight, as previously reported [35]. In short, the medium was gradually transitioned to Neural Induction Medium 1 (NIM1). The newly formed embryoid bodies (EBs) were seeded on Matrigel-coated 6-wells plates in NIM1. Smoothened agonist (SAG) (Selleck Chemicals, Houston, TX, USA) (100 nM) was added from DD10, and from DD16, the EBs were cultured in Neural Induction Medium 2 (NIM2) with SAG. Between DD21 and DD28, neuroepithelial structures were mechanically lifted and cultured on agarose-coated 48-well plates with NIM2. From DD35 to DD44, organoids were fed with Retinal Lamination Medium 1 (RLM1) + 1 µM of retinoic acid (Merck, Schiphol-Rijk, The Netherlands), then RLM1 + 1 µM of retinoic acid + 10 µM of gamma secretase inhibitor IX (DAPT) (Selleck Chemicals, Houston, TX, USA) until DD55. From DD56, organoids were cultured with RLM1 + 1 µM of retinoic acid until DD85, then switched to Retinal Lamination Medium 2 (RLM2) + 0.5 µM of retinoic acid. From DD120, RLM2 alone was used until collection. Medium composition and materials are detailed in the Supplementary Methods and Table S3.

### 4.3. Immunohistochemical Analysis

Retinal organoids at DD225 were fixed with 4% paraformaldehyde in PBS for 20 min at RT, rinsed with PBS for 1 min, and subsequently incubated with 15% sucrose in PBS for 30 min and with 30% sucrose in PBS for at least 1 h, until organoids sank to the bottom of the well. Thereafter, they were embedded in Tissue-Tek O.C.T. Compound (Sakura Finetek Europe, Alphen aan den Rijn, The Netherlands) and sliced into 8 µm cryosections using the Leica CM1900 cryostat (Leica Microsystems, Wetzlar, Germany). Slides were stored at −20 °C until use. Immunohistochemical analysis was performed as previously described [29]. Three images were taken per organoid with 40× and 100× magnification. Information related to the materials and antibodies used in this study are reported in Tables S3 and S4. The detailed protocol can be consulted in the Supplementary Methods.

### 4.4. Quantification and Statistical Analysis

Quantification was performed using Fiji ImageJ version 2.17. The outer nuclear layer (ONL) thickness was manually quantified from three independent regions in each image. For the ARL13 length quantification, the length of all photoreceptor cilia in the image was measured and then averaged per organoid. For the rhodopsin distribution between the ONL and the photoreceptor segments, two ROIs were drawn following the F-actin staining at the outer limiting membrane, and the intensity for each ROI was measured and expressed as a fraction of the total intensity. For the GFAP and CD44 IHC quantification, intensity was measured per field of view. For the ROM1 quantification, intensity per field of view was measured and plotted with set cut-off value of 50,000,000. Graphs were generated using GraphPad Prism version 10.2.3, where each datapoint corresponds to an individual organoid and results from the average of three independent images. Data are presented as mean per organoid ± standard error of the mean. Normality was assessed using the Shapiro–Wilk test. Data distribution was visually inspected using QQ plots. Significance was calculated by one-way ANOVA followed by Dunnett’s multiple comparisons test and indicated as *p* < 0.05 (*), *p* < 0.01 (**), *p* < 0.001 (***), and ns (not significant). When data violated normality assumptions, group differences were assessed using the Kruskal–Wallis test followed by Dunn’s multiple comparisons test. Four batches of differentiation for ISO-CTRL, 13KO E11, and 13KO G8, and three batches of differentiation for 13KO B7 were analyzed to confirm the phenotype by immunohistochemistry.

### 4.5. Single-Cell RNA Sequencing

Single-cell RNA sequencing data were generated using Chromium 10X 3′UTR-sequencing. Number of organoids used: ISO-CTRL *n* = 4, 13KO E11 *n* = 4, 13KO G8 *n* = 3, and 13KO B7 *n* = 3 from the same differentiation round. Fourteen individual retinal organoids were used for 14 scRNA-Seq. Once the organoids were dissociated into single cells using the Papain Dissociation kit (Worthington Biochemical Corp., Lakewood, NJ, USA), the suspensions were loaded onto the Chromium Single Cell system using the v3 chemistry. Subsequent steps were performed according to the manufacturer’s instructions. Cell Ranger (v7.1.0) pipeline (10X Genomics) was used to process raw sequencing output with the default human genome reference GRCh38. Filtered expression matrices were further processed with a Seurat-based workflow in R (v4.4.0). Differential expression at the single-cell level was assessed using Wilcoxon rank-sum tests as implemented in Seurat, with *p*-values adjusted for multiple testing using the Benjamini–Hochberg false-discovery rate (FDR). Genes with FDR < 0.05 were considered differentially expressed. Detailed downstream analysis is described in the Supplementary Methods.

## Figures and Tables

**Table 1:** List of patient and 13KO *USH2A* mutations resulting in a similar truncated usherin protein structure.

*USH2A* Mutation	Usherin Change	Usherin Size (aa)	Protein Domain Affected
c.2311 G>T	p.(Glu771Ter)	770	EGF-Lam like 5
c.2299del	p.(Glu767Serfs*21)	786 (766 + 20)	EGF-Lam like 5
c.2310del	p.(Glu771Lysfs*17)	786 (770 + 16)	EGF-Lam like 5
c.2314delG	p.(Ala772Profs*16)	786 (771 + 15)	EGF-Lam like 5
c.2344del	p.Glu782Lysfs*6)	786 (781 + 5)	EGF-Lam like 5
c.2391_2392del	p.(Cys797*)	796	EGF-Lam like 6
c.2431 A>T	p.(Lys811*)	810	EGF-Lam like 6
c.2431_2432_del	p.(Lys811Aspfs*11)	820 (810 + 10)	EGF-Lam like 6
c.2440 C>T	p.(Gln814*)	813	EGF-Lam like 6
c.2512 C>T	p.(Glu838*)	837	EGF-Lam like 6
c.2541 C>A	p.(Cys847*)	846	EGF-Lam like 7
**c.2553_2554insTAGT**	p.(Thr852*)	**851**	**EGF-Lam like 7**
c.2599 C>T	p.(Gln867*)	866	EGF-Lam like 7
c.2610 C>A	p.(Cys870*)	869	EGF-Lam like 7
C.2661 C>A	p.(Tyr887)	886	EGF-Lam like 7

The information related to the *USH2A* 13KO mutation generated in this study is reported in bold for reference. In case of frameshift mutations, the number of nucleotides differing from the original usherin sequence is given in brackets. The identity of the protein domain affected is determined following the NCBI annotation.

## Data Availability

The data that support the findings of this study are available from the corresponding author upon reasonable request. scRNA-Seq data are openly available in the NCBI Gene Expression Omnibus database (GEO: GSE290344).

## Supplementary Materials

The following supporting information can be downloaded at: https://www.mdpi.com/article/10.3390/ijms27041636/s1.

## Abbreviations

The following abbreviations are used in this manuscript:

AAV	Adeno-associated viral vectors
DD	Differentiation day
DEG	Differentially expressed gene
MGCs	Müller glial cells
ONL	Outer nuclear layer
TEM	Transmission electron microscopy
UMAP	Uniform Manifold Approximation and Projection
