# Supplementary material for: Nonsense Mutation in USH2A Exon-13 Activates the Innate Immune Response in Müller Glial Cells
Source: Int J Mol Sci. 2026 Feb 7;27(4):1636. doi: 10.3390/ijms27041636 (PMC12940303; doi:10.3390/ijms27041636)
Supplement: Supplementary file 1 [file ijms-27-01636-s001.zip › ijms-4039014-supplementary_revised_06022026.pdf]

Nonsense mutation in *USH2A* exon-13 activates the innate immune response in Müller glial cells

Rossella Valenzano [1], Xuefei Lu [1], Andrew McDonald [1], Ioannis Moustakas [2], Roberta Menafra [3], Aat Mulder [4], Roman I. Koning [4], Susan Kloet [3], Jun Yang [5], Hailiang Mei [2] and Jan Wijnholds [1*]

Supplementary Materials

A.

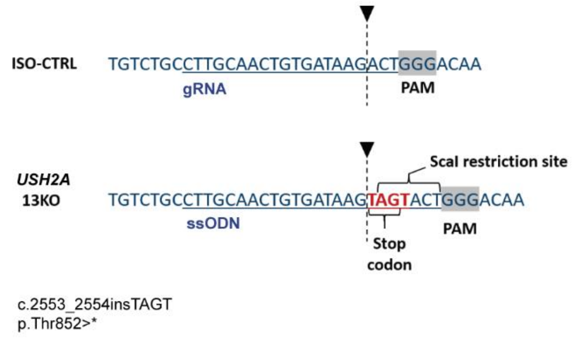

B.

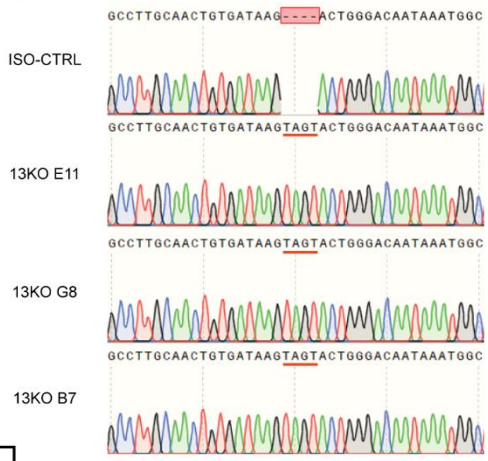

C.

Summary of the detected copy number variations																						
Chromosome	1p	1q	2q	3p	4q	5q	6q	7p	8q	9q	11p	12p	13q	14q	15q	16q	17p	18q	19p	20q	22q	Xp
ISO-CTRL																						
13KO E11																						
13KO G8																						
13KO B7																						

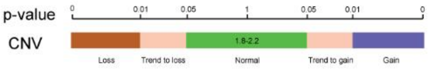

D.

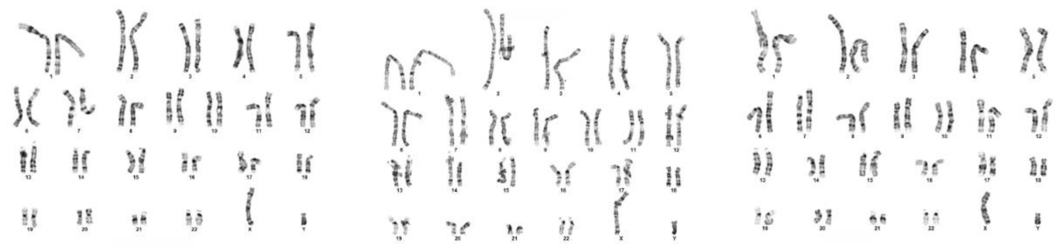

E.

	Short tandem repeat profile										
Markers	CSF1PO	D1S1656	D2S1338	D2S441	D3S1358	D5S818	D7S820	D8S1179	D10S1248	D12S391	D13S317
ISO-CTRL	11	16.3,17.3	19,20	12,14	17,18	12,13	9,10	14	13,15	19,22	11,13
13KO E11	11	16.3,17.3	19,20	12,14	17,18	12,13	9,10	14	13,15	19,22	11,13
13KO G8	11	16.3,17.3	19,20	12,14	17,18	12,13	9,10	14	13,15	19,22	11,13
13KO B7	11	16.3,17.3	19,20	12,14	17,18	12,13	9,10	14	13,15	19,22	11,13
Markers	D16S539	D18S51	D19S433	D21S11	D22S1045	FGA	Penta D	Penta E	TH01	TPOX	vWA
ISO-CTRL	8,15	13	12	27,30	16	24	10,12	5,12	7,10	8	16,18
13KO E11	8,15	13	12	27,30	16	24	10,12	5,12	7,10	8	16,18
13KO G8	8,15	13	12	27,30	16	24	10,12	5,12	7,10	8	16,18
13KO B7	8,15	13	12	27,30	16	24	10,12	5,12	7,10	8	16,18

Figure S1: CRISPR/Cas9-mediated generation of *USH2A* 13KO hiPSCs and genotypic analyses. (**A**) Schematics illustrating the KO design, with an ssODN (single-stranded oligodeoxynucleotide) donor

mediating the insertion of a premature stop codon and a nucleotide frameshift 3 bp upstream the PAM of the gRNA of choice in *USH2A* exon-13. The correct insertion recreates a ScaI restriction site necessary for the colony screening upon SpCas9/gRNA delivery in the parental cell line. (**B**) Confirmation of the homozygous *USH2A* 13KO hiPSCs generation via Sanger sequencing. (**C**) Analysis of the most recurrent abnormalities, showing no detection of copy number variations (CNVs) in the *USH2A* 13KO hiPSC subclones. (**D**) Karyotyping results of *USH2A* 13KO hiPSC subclones, showing normal metaphases. (**E**) Short tandem repeat profiling confirming the derivation of all *USH2A* 13KO hiPSC subclones from the same parental cell line, ISO-CTRL.

A.

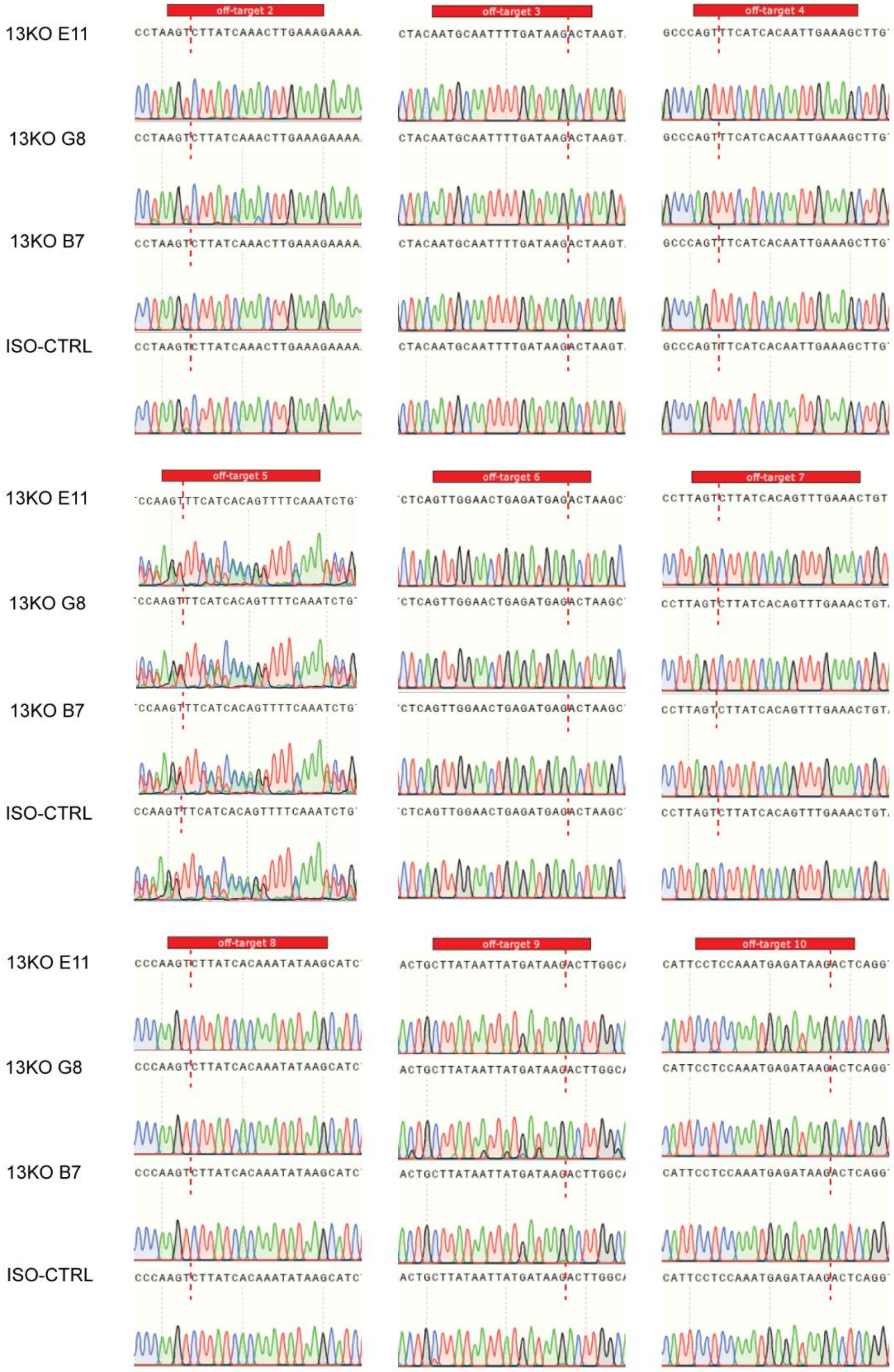
13KO E11
13KO G8
13KO B7
ISO-CTRL
off-target 2
off-target 3
off-target 4
off-target 5
off-target 6
off-target 7
off-target 8
off-target 9
off-target 10

B.

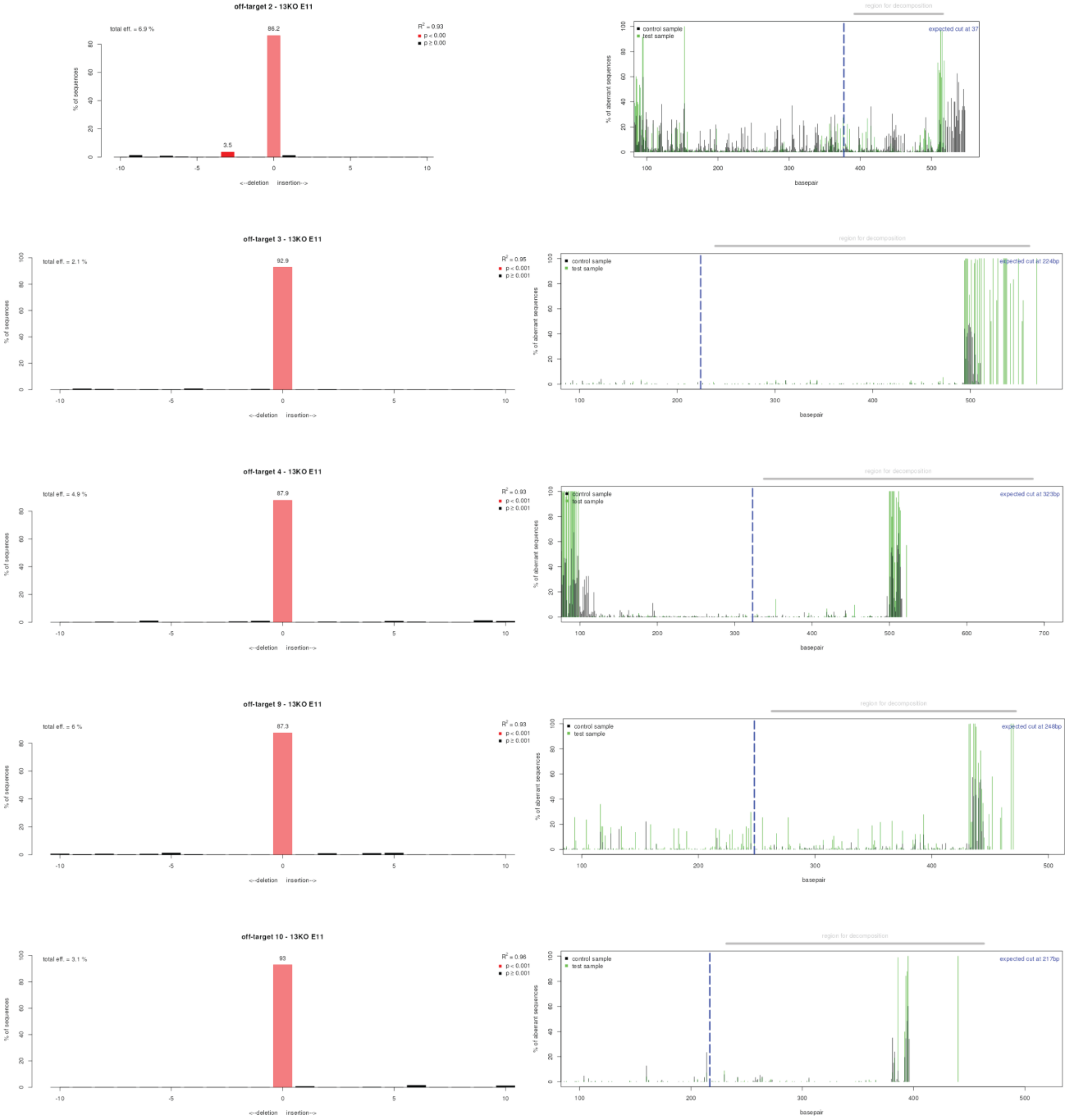
off-target 2 - 13KO E11
total eff. = 6.9 %
86.2
3.5
<-deletion insertion-->
% of sequences
region for decomposition
control sample
test sample
% of aberrant sequences
basepair
off-target 3 - 13KO E11
total eff. = 2.1 %
92.9
expected cut at 224bp
off-target 4 - 13KO E11
total eff. = 4.9 %
87.9
expected cut at 323bp
off-target 9 - 13KO E11
total eff. = 6 %
87.3
expected cut at 248bp
off-target 10 - 13KO E11
total eff. = 3.1 %
93
expected cut at 217bp

C.

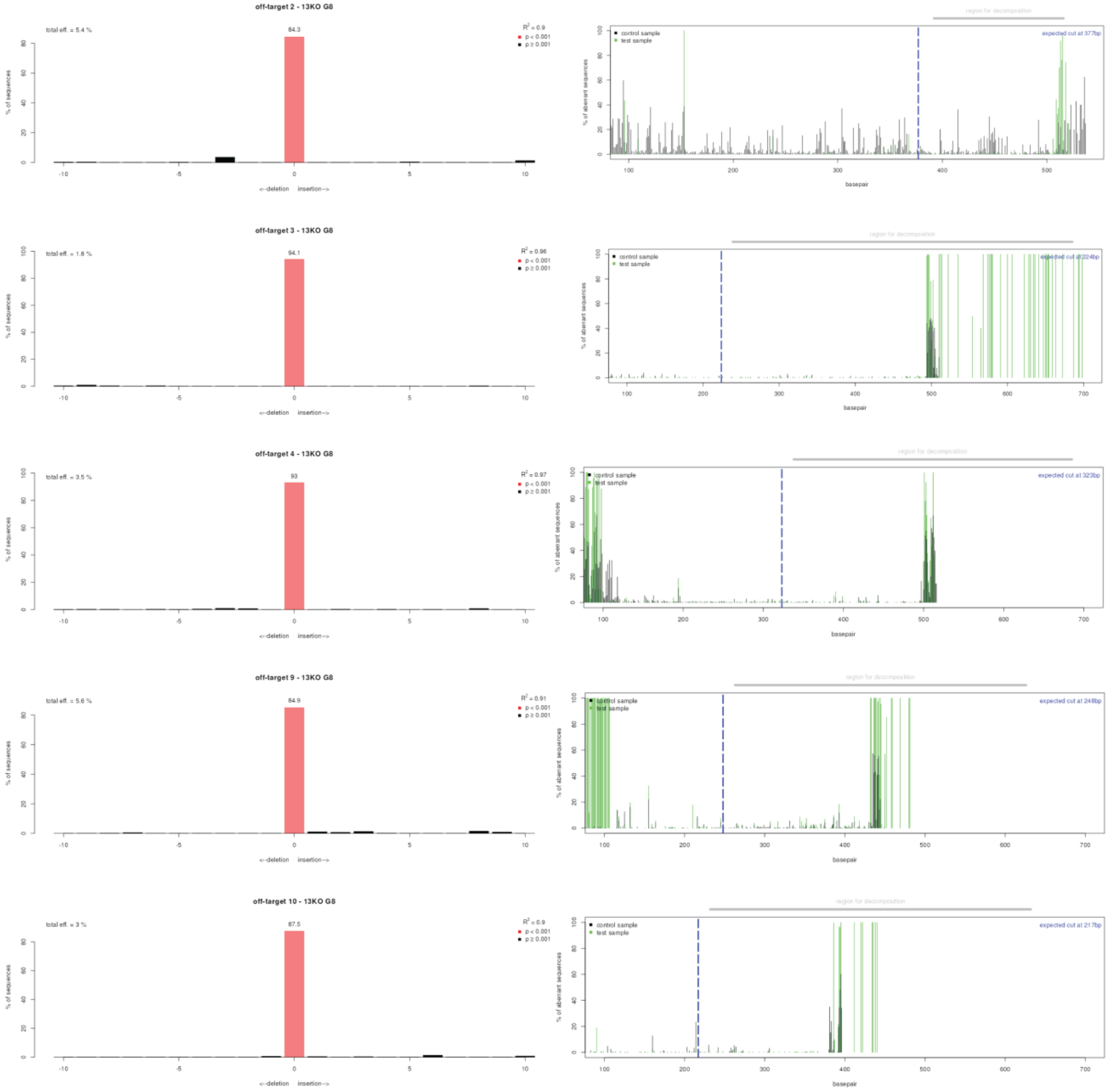
off-target 2 - 13KO G8
total eff. = 5.4 %
84.3
R^2 = 0.9
p < 0.001
p ≥ 0.001
% of sequences
<-deletion insertion->
region for decomposition
control sample
test sample
expected cut at 377bp
% of aberrant sequences
basepair
off-target 3 - 13KO G8
total eff. = 1.8 %
94.1
R^2 = 0.96
expected cut at 224bp
off-target 4 - 13KO G8
total eff. = 3.5 %
93
R^2 = 0.97
expected cut at 323bp
off-target 9 - 13KO G8
total eff. = 5.6 %
84.9
R^2 = 0.91
expected cut at 248bp
off-target 10 - 13KO G8
total eff. = 3 %
87.5
R^2 = 0.9
expected cut at 217bp

D.

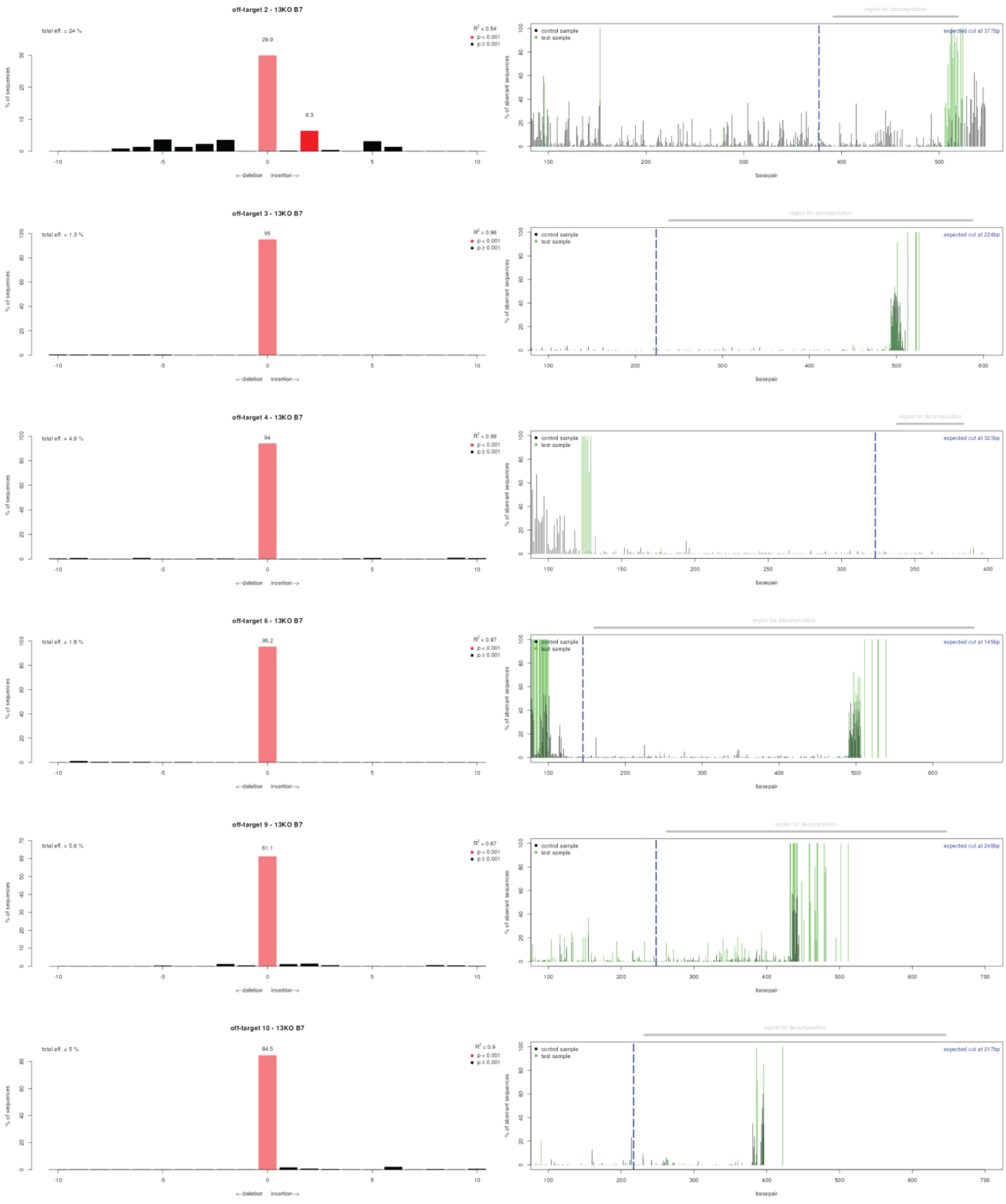
off-target 2 - 13KO B7
total eff. = 24 %
R^2 = 0.54
p < 0.001
p ≥ 0.001
29.9
6.3
% of sequences
<--deletion insertion-->
region for decomposition
control sample
test sample
expected cut at 377bp
% of aberrant sequences
basepair
off-target 3 - 13KO B7
total eff. = 1.3 %
R^2 = 0.96
95
expected cut at 224bp
off-target 4 - 13KO B7
total eff. = 4.6 %
R^2 = 0.99
94
expected cut at 323bp
off-target 6 - 13KO B7
total eff. = 1.8 %
R^2 = 0.97
95.2
expected cut at 145bp
off-target 9 - 13KO B7
total eff. = 5.6 %
R^2 = 0.67
61.1
expected cut at 248bp
off-target 10 - 13KO B7
total eff. = 5 %
R^2 = 0.9
84.5
expected cut at 217bp

Figure S2: Off-target mutagenesis analysis of *USH2A* 13KO hiPSC subclones. (**A**) Analysis of the top 10 off-target sites associated with the gRNA used to generate the *USH2A* 13KO hiPSC subclones by Sanger sequencing. Off-target hits were predicted by IDT software using the "CRISPR-Cas9 guide RNA design checker" tool and attributed a score from 1 to 100, where a lower value indicates higher off-target potential editing. Off-target 1: score 8; off-target 2: score 9; off-target 3: score 9; off-target 4: score 13; off-target 5: score 13; off-target 6: score 14; off-target 7: score 18; off-target 8: score 18; off-target 9: score 19; off-target 10: score 23. Sequencing of off-target site 1 was not successful. Off-target hit 1 (chr6:-26266634) resides in a unique 162 bp region of 5′-GTTATTTTCTTTTCCC<u>AGTCTTATCTCAGTTGTGAG</u>TTCTTCCCCATCCACCATTTTTAAAATTACAGCTGTCTACCACATTCCCTCCTGTCCTCTGGCAGTCATTCCCTTTATCCATTTTAACTTTTCTACGTCTTTTTTATATAAGAATATACTAAACTC-3′ flanked by a stretch of 14 A-nucleotides and a stretch of 18 T-nucleotides at the 5′ and 3′ ends, respectively. Moreover, additional repetitive sequences are present both immediately upstream and downstream of the entire region of interest. Despite multiple attempts, we were not successful in cloning and subsequent sequencing of off-target site 1 using genomic DNA from isogenic control and mutant *USH2A* 13KO E11, G8, and B7 cell lines. However, the other nine sites (off-targets 2-10) were successfully sequenced. The expected cut site is indicated with a red dashed line. (**B–D**) Tracking of indels by decomposition (TIDE) in the 13KO E11 (**B**), 13KO G8 (**C**), and 13KO B7 (**D**) cell lines. The indel spectrum is flanked by the quality control–aberrant sequence signal. Some of the off-targets could not be analyzed due to insufficient width of the alignment window between the control and test sample. TIDE analysis detected overall low-level indel formation, ranging from 2.1% to 6.9% for 13KO E11 (**B**), from 1.8% to 5.6% for 13KO G8 (**C**), and from 1.3% to 24% for 13KO B7 (**D**). Although the total editing rate at off-target site 2 reached 24% in 13KO B7, this should not be considered alarming: the TIDE indel spectrum revealed a heterogeneous distribution of indels from -7 bp to +6 bp, with the largest peak at 6.3% for a +2 bp indel (**D**). Such a diffuse indel profile is consistent with low-level off-target activity.

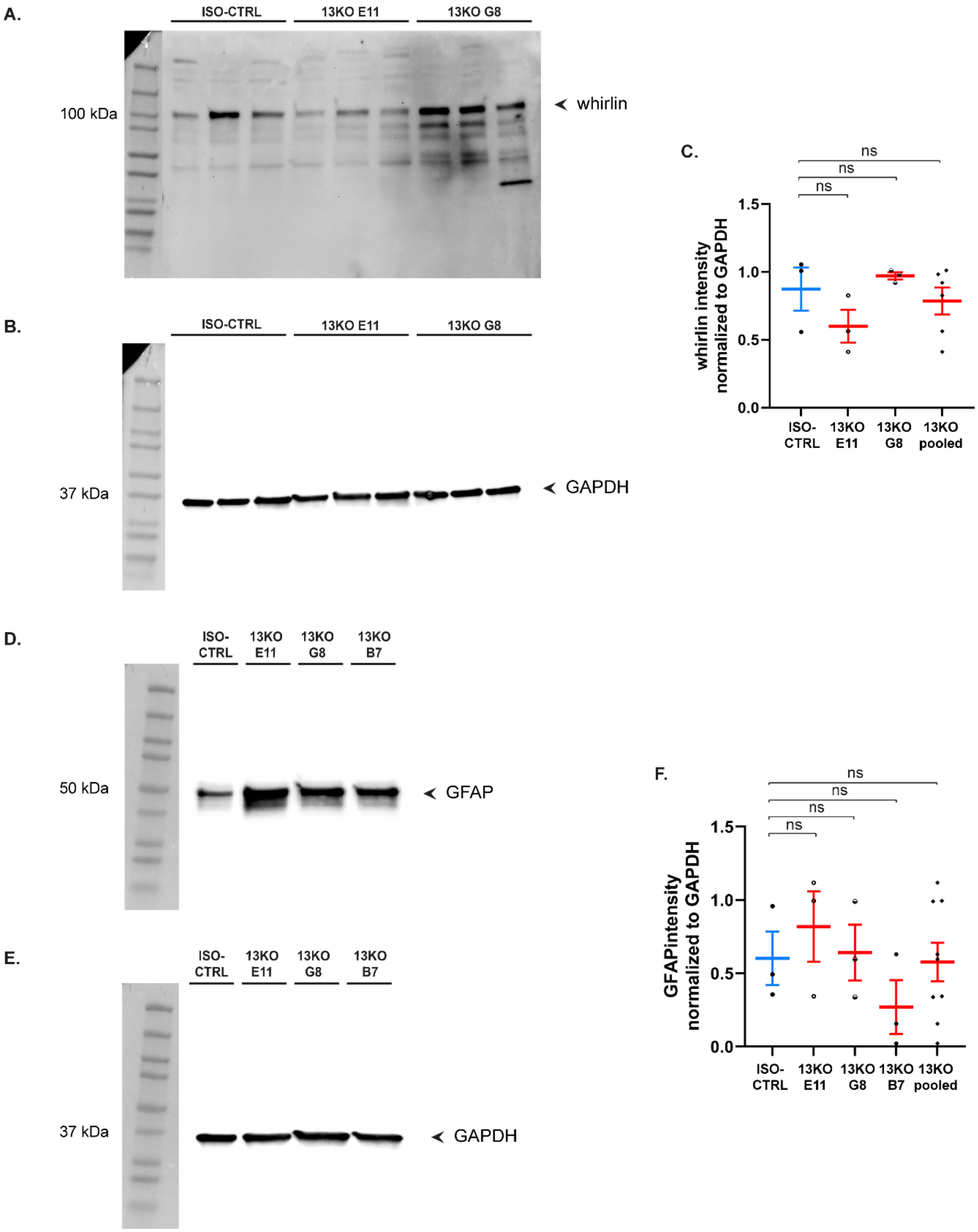

Figure S3: Western Blot analysis of whirlin and GFAP in *USH2A* 13KO retinal organoids. (**A, B**) Detection of whirlin (**A**) and glyceraldehyde 3-phosphate dehydrogenase (GADPH) (**B**) in lysates from ISO-CTRL

and two 13KO subclones. In each lane, 16 μg of protein lysate in a final volume of 40 μl was loaded. (**C**) Quantitative analysis of whirlin intensity normalized to GAPDH intensity. Number of organoids used: ISO-CTRL $n = 3$, 13KO E11 $n = 3$, 13KO G8 $n = 3$, and 13KO pooled $n = 6$, from the same differentiation round. Given the small sample size, normality could not be reliably assessed; therefore nonparametric tests were used. Significance was calculated by the Kruskal–Wallis tast followed by Dunn's multiple comparisons test. Statistical analysis: $p = 0.347$, $p > 0.99$, and $p > 0.99$, from left to right. (**D, E**) Detection of GFAP (glial fibrillary acidic protein) (**D**) and GAPDH (**E**) in lysates from ISO-CTRL and three 13KO subclones. In each lane, 20 μg of protein lysate in a final volume of 40 μl was loaded. (**F**) Quantitative analysis of GFAP intensity normalized to GAPDH intensity. Number of organoids used: ISO-CTRL $n = 3$, 13KO E11 $n = 3$, 13KO G8 $n = 3$, 13KO B7 $n = 3$, and 13KO pooled $n = 9$, from the same differentiation round. Given the small sample size, normality could not be reliably assessed; therefore nonparametric tests were used. Significance was calculated by the Kruskal–Wallis test followed by Dunn's multiple comparisons test. Statistical analysis: $p > 0.99$, $p > 0.99$, $p = > 0.99$, and $p > 0.99$, from left to right.

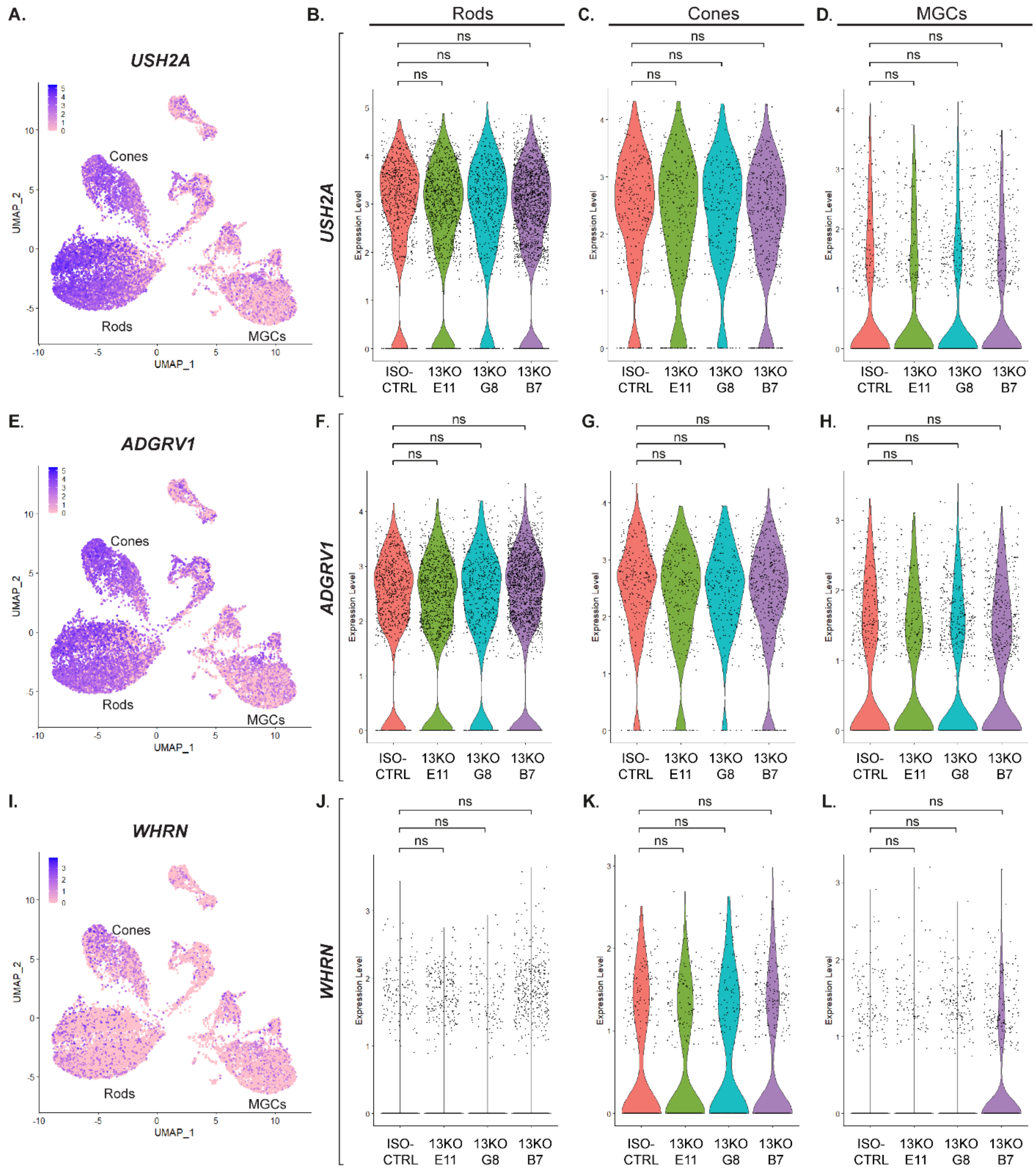

Figure S4. Expression of *USH2A*, *ADGRV1*, and *WHRN* genes in photoreceptor and Müller glial cells. (**A, E, I**) UMAP plots showing expression of *USH2A* (**A**), *ADGRV1* (**E**), and *WHRN* (**I**) across cell clusters in retinal organoids. (**B, C, D**) Violin plots comparing *USH2A* expression levels in the 13KO subclones to ISO-

CTRL in rods (**A**), cones (**C**), and MGCs (**D**) (adjusted p-values = 1). (**F, G, H**) Violin plots comparing *ADGRV1* expression levels in the 13KO subclones to ISO-CTRL in rods (**F**), cones (**G**), and MGCs (**H**) (adjusted p-values = 1). (**J, K, L**) Violin plots comparing *WHRN* expression levels in the 13KO subclones to ISO-CTRL in rods (**J**), cones (**K**), and MGCs (**L**) (adjusted p-values = 1). Number of organoids used: ISO-CTRL *n* = 4, 13KO E11 *n* = 4, 13KO G8 *n* = 3, and 13KO B7 *n* = 3, from the same differentiation round. Differential expression at the single-cell level was assessed using Wilcoxon rank-sum tests as implemented in Seurat, with p-values adjusted for multiple testing using the Benjamini–Hochberg false-discovery rate (FDR). Genes with FDR < 0.05 were considered differentially expressed.

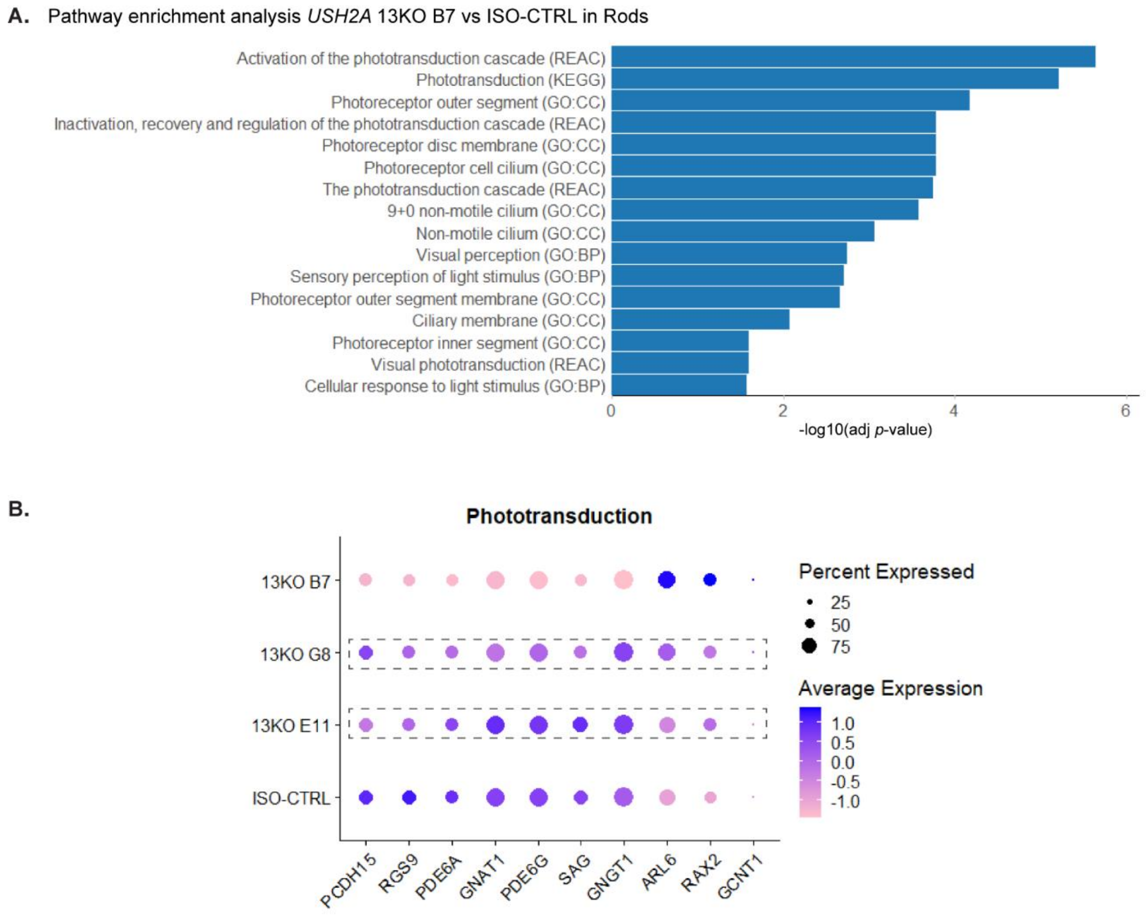

Figure S5. Disruption of phototransduction is detected in the rod photoreceptors of *USH2A* 13KO subclone B7. (**A**) Pathway enrichment analysis of differentially expressed genes (DEGs) exclusively in the rods of subclone *USH2A* 13KO subclone B7, but not of *USH2A* 13KO E11 and *USH2A* 13KO G8, compared to isogenic controls, and related to the cellular components and biological process of phototransduction. (**B**) Statistically significant DEGs in *USH2A* 13KO subclone B7 associated with the phototransduction category terms. Dashed boxes indicate absence of differential expression compared to ISO-CTRL. Number of organoids used: ISO-CTRL n = 4, 13KO E11 n = 4, 13KO G8 n = 3, and 13KO B7 n = 3 from the same differentiation round. Differential expression at the single-cell level was assessed using Wilcoxon rank-sum tests as implemented in Seurat, with p-values adjusted for multiple testing using the Benjamini–Hochberg false-discovery rate (FDR). Genes with FDR < 0.05 were considered differentially expressed.

A. Volcano plots of *USH2A* 13KO sublones vs ISO-CTRL Rods

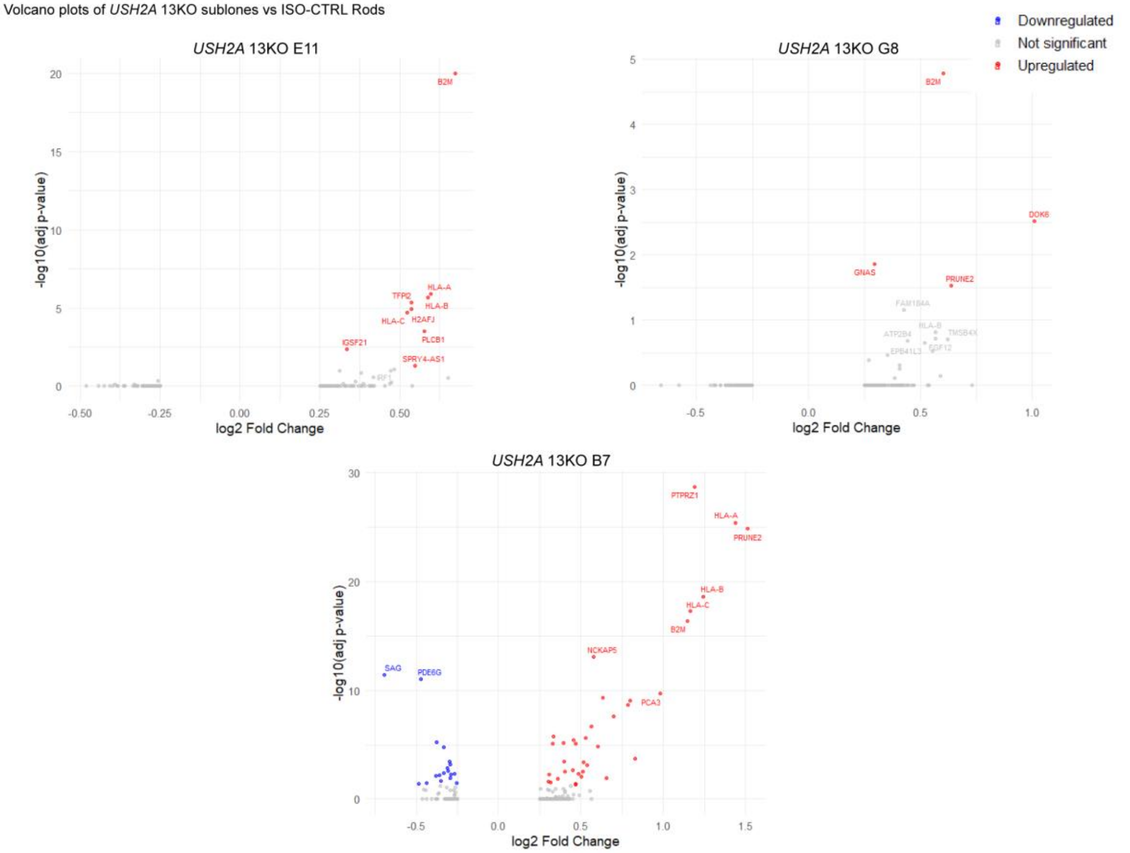

B. Volcano plots of *USH2A* 13KO sublones vs ISO-CTRL MGCs

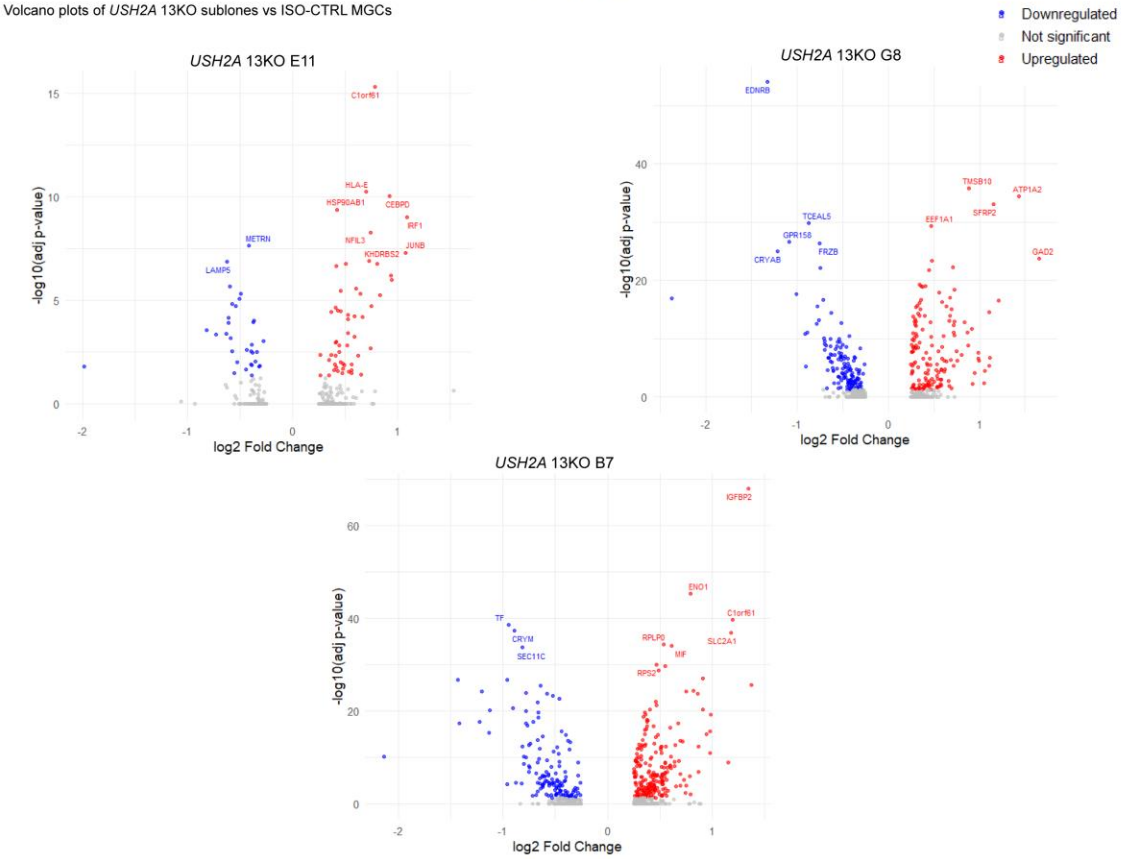

Figure S6. Volcano plots showing differentially expressed genes in rods and MGCs of *USH2A* 13KO subclones. (**A–B**) Volcano plots highlighting the upregulated (red) and downregulated (blue) genes in the rod photoreceptors (**A**) and MGCs (**B**) of each *USH2A* 13KO subclone when compared to ISO-CTRL. Statistical parameters: Wilcoxon test, adjusted p value < 0.05, absolute log2 fold change threshold = 0.25. The top 10 DEGs are indicated in each volcano plot. Number of organoids used: ISO-CTRL $n = 4$, 13KO E11 $n = 4$, 13KO G8 $n = 3$, and 13KO B7 $n = 3$, from the same differentiation round.

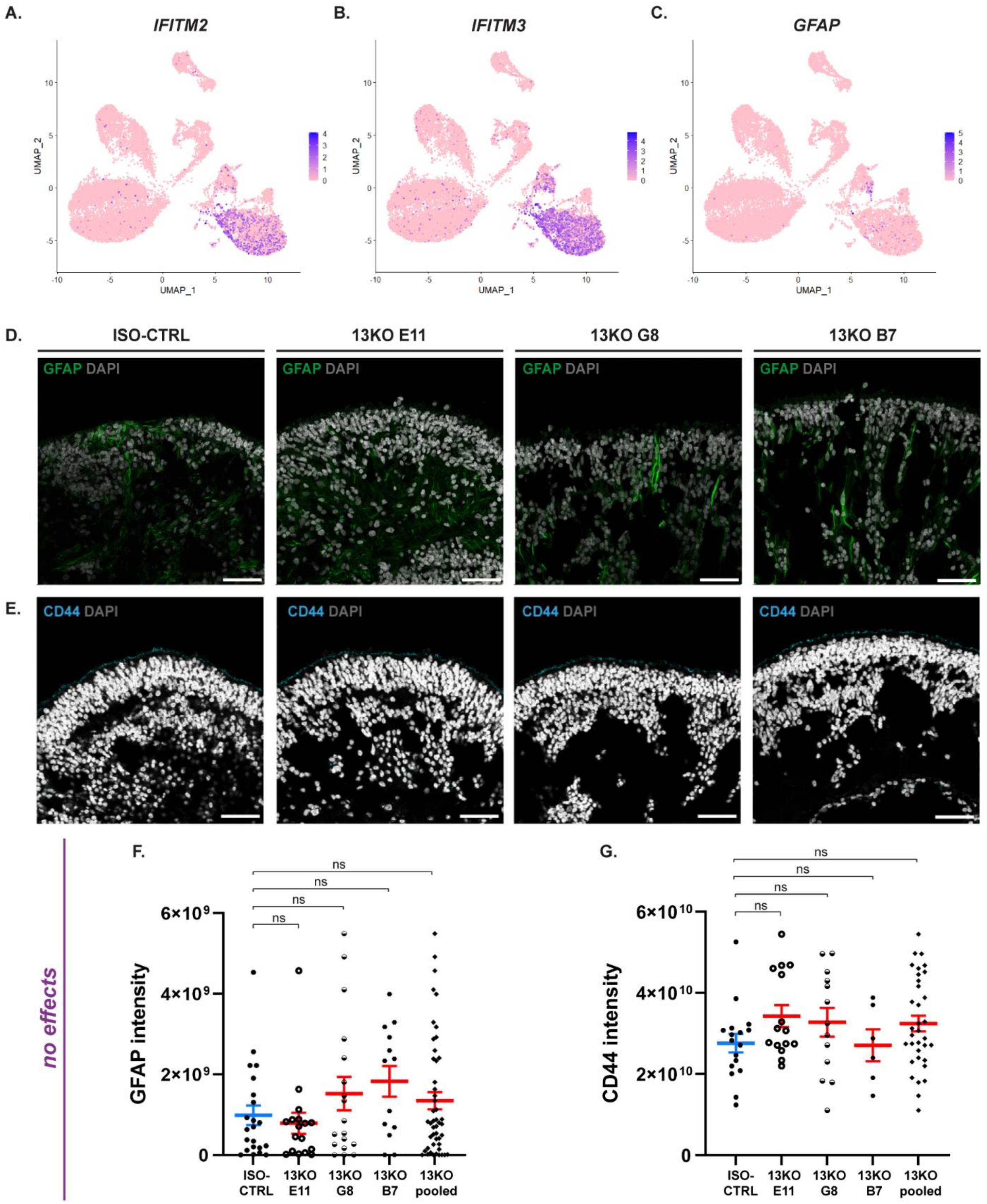

Figure S7: Characterization of Müller glial cells in retinal organoids upon *USH2A* loss. (**A, B, C**) UMAP plots displaying expression of *IFITM2* (**A**), *IFITM3* (**B**), and *GFAP* (**C**) across cell clusters in retinal

organoids. (**D, E**) Representative immunohistochemical images of MGCs markers, GFAP (**D**, green, Dako Z0334 antibody, 1:400 dilution) and CD44 (**E**, blue, Thermo Fisher Scientific MA4400, 1:300 dilution). Images in (**D**) are Z-stack views; images in (**E**) are single-plane views. Scale bars = 50 µm. (**F**) Quantitative analysis of GFAP intensity. Number of organoids used: ISO-CTRL $n = 22$, 13KO E11 $n = 17$, and 13KO G8 $n = 18$, from four rounds of differentiation, and 13KO B7 $n = 13$, from three rounds of differentiation; 13KO pooled $n = 48$. Normality was assessed using the Shapiro–Wilk test combined with visual inspection of data distribution via QQ plots. As data did not meet normality assumptions, a nonparametric test was used. Significance was calculated by the Kruskal–Wallis test followed by Dunn's multiple comparisons test. Statistical analysis: $p > 0.99$, $p > 0.99$, $p = 0.36$, and $p > 0.99$, from left to right. (**G**) Quantitative analysis of CD44 intensity. Number of organoids used: ISO-CTRL $n = 17$, 13KO E11 $n = 15$, and 13KO G8 $n = 13$, from four rounds of differentiation, and 13KO B7 $n = 6$, from three rounds of differentiation; 13KO pooled $n = 34$. Normality was assessed using the Shapiro–Wilk test combined with visual inspection of data distribution via QQ plots. Significance was calculated by one-way ANOVA followed by Dunnett's multiple comparisons test. Statistical analysis: $p = 0.263$, $p = 0.522$, $p = 0.999$, and $p = 0.391$, from left to right. Purple side bars highlight the absence of effects resulting from the KO.

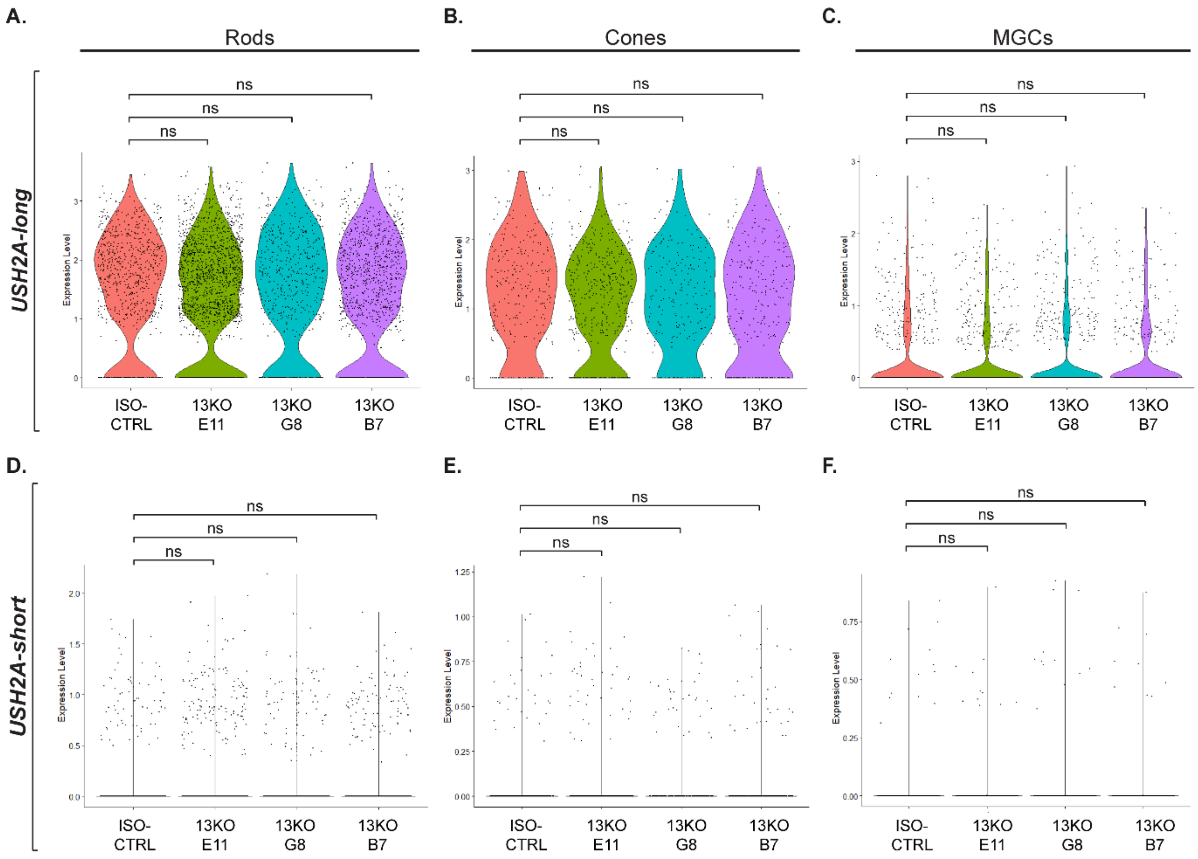

Figure S8. Analysis of *USH2A* transcripts coding for the long and short usherin isoforms in photoreceptors and Müller glial cells of *USH2A* 13KO retinal organoids compared to isogenic controls. (**A–F**) Violin plots comparing the expression levels of *USH2A* coding for usherin long isoform B (**A–C**) and usherin short isoform A (**D–F**) in the 13KO subclones to ISO-CTRL in rods (**A, D**), cones (**B, E**), and MGCs (**C, F**) (adjusted p-values = 1). Number of organoids used: ISO-CTRL $n = 4$, 13KO E11 $n = 4$, 13KO G8 $n = 3$, and 13KO B7 $n = 3$ from the same differentiation round. Differential expression at the single-cell level was assessed using Wilcoxon rank-sum tests as implemented in Seurat, with p-values adjusted for multiple testing using the Benjamini–Hochberg false-discovery rate (FDR). Genes with FDR < 0.05 were considered differentially expressed.

Table S1: hiPSC line information.

Line name	Description	Gender
**LUMC0004iCTRL10 = ISO-CTRL (hPSCreg name: LUMCi029-B)**	Control parental hiPSC line	Male
**LUMC0004iCTRL10_*USH2A*^{13KO} CLE11 = 13KO E11**	It has a stop codon in exon-13 of *USH2A*. c.2553_2554insTAGT, p.(Thr852*)	Male
**LUMC0004iCTRL10_*USH2A*^{13KO} CLG8 = 13KO G8**	It has a stop codon in exon-13 of *USH2A*. c.2553_2554insTAGT, p.(Thr852*)	Male
**LUMC0004iCTRL10_*USH2A*^{13KO} CLB7 = 13KO B7**	It has a stop codon in exon-13 of *USH2A*. c.2553_2554insTAGT, p.(Thr852*)	Male

Information on the hiPSC lines used in this study.

Table S2: Information related to the crRNA and donor template used to generate the *USH2A* 13KO hiPSC lines and primers to assess the correct genomic mutation and absence of off-target mutagenesis.

Name	Sequence (5′-3′)
***USH2A* exon-13 crRNA**	CTTGCAACTGTGATAAGACT
***USH2A* ssODN**	/AlT-R-HDR1/T*G*TTGAAGGGAGACAGTGCAATAAATGTTTGGAGGGAAACTTCTACCTACGGCAAAATAATTCTTTCCTCTGTCTGCCTTGCAACTGTGATAAGTAGTACTGGGACAATAAATGGCTCTCTGCTGTGTAACAAATCAACAGGACAATGTCCTTGCAAATTAGGGGTAACAGGTCTTCGCTGTAATCAGTG*T*G/AlT-R-HDR2/
***USH2A* exon-13 Fw primer**	CCTCCCTGGGACTGTCTGTA
***USH2A* exon-13 Rv primer**	GCCACAAACCAGAAACAGGG
**Off-target 1 (chr6:-26266634) Fw primer**	TTTTCTTGGCCTGGTGTGGT
**Off-target 1 (chr6:-26266634) Rv primer**	TTCGAGACCAGTTTGGCCAA
**Off-target 2 (chr6:-64818159) Fw primer**	TTTTATGGCCTCCAGCTGCA
**Off-target 2 (chr6:-64818159) Rv primer**	AAATGCATGCCTGGGGAGAG
**Off-target 3 (chr5:+38980446) Fw primer**	CTGAATCACCTGCAAGCATGT
**Off-target 3 (chr5:+38980446) Rv primer**	CCTCCTGAGTAGCTGGGACT
**Off-target 4 (chr13:-79524955) Fw primer**	AGGATGGTCAGCTGCATTGT
**Off-target 4 (chr13:-79524955) Rv primer**	AGACACTGAGCCATTTACTGCA
**Off-target 5 (chr6:-58201278) Fw primer**	GAGTGGGCCAGGTAAAAGGT

**Off-target 5 (chr6:-58201278) Rv primer**	TCTGTTGTTACTGCCAGCACA
**Off-target 6 (chr14:+66122003) Fw primer**	TGTTTTCCCCGTGCCCATAT
**Off-target 6 (chr14:+66122003) Rv primer**	CTAGCAAGGAGAGGCACCAG
**Off-target 7 (chr7:-91294366) Fw primer**	TCTGCAAAAGTGGGTGAGGT
**Off-target 7 (chr7:-91294366) Rv primer**	AGAATGGTCCCAAGCTGCAA
**Off-target 8 (chr15:-95927540) Fw primer**	GCACTCAGAAGCACTTGTCC
**Off-target 8 (chr15:-95927540) Rv primer**	CCTGCTGGCACCTTGATCTT
**Off-target 9 (chr22:+32852014) Fwprimer**	GATCCCCAAGCTGAGGCATC
**Off-target 9 (chr22:+32852014) Rv primer**	GTTTTGCTCAGGTTTGCCACT
**Off-target 10 (chr6:+158878904) Fw primer**	GCCAGGCCTAGTTTCCTTTTTC
**Off-target 10 (chr6:+158878904) Rv primer**	TTAGAGGGGCCCCACACTTT

Forward (Fw) and reverse (Rv) primers used to confirm the CRISPR/Cas9-mediated insertion of TAGT in *USH2A* exon-13 and the absence of the top 10 predicted off-targets.

Table S3: List of materials used in this study.

Materials	Source	Identifier
**tracrRNA**	IDT	1072532
**Nuclease-Free Duplex Buffer**	IDT	11-01-03-01
**SpCas9 Nuclease V3**	IDT	1081058
**Neon™ Transfection System kit**	Invitrogen	MPK10096
**Neon™ Transfection System**	Invitrogen	MPK1025
**Matrigel hESC-Qualified Matrix**	Corning	354277
**mTeSR plus medium**	STEMCELL Technologies	100-0276
**Accumax**	STEMCELL Technologies	07921
**Gentle Cell Dissociation reagent**	STEMCELL Technologies	100-0485
**CloneR**	STEMCELL Technologies	05888
**Cryostor**	STEMCELL Technologies	07930
**DMEM/F12**	Life Technologies	11320074
**DMEM(1X) + GlutaMAX**	ThermoScientific	10569010
**Fasudil HCl**	Focus Biomolecules	10-2137
**40 µm cell strainer**	pluriSelect	43-10040-40
**ScaI**	ThermoScientific	ER0431
**Blebbistatin**	abcam	ab120425
**Micro-molds**	Merck	Z764000-6EA
**MEM NEAA 100X**	Life technologies	11140-035
**Taurine**	Merck	T0625
**Neurocult SM1 50X**	STEMCELL Technologies	05711

**N2 supplement 100X**	Life technologies	17502048
**Heparin**	Merck	H-9399
**Smoothened agonist (SAG)**	Selleck Chemicals	S7779
**Gamma secretase inhibitor IX (DAPT)**	Selleck Chemicals	S2215
**Fetal Bovine Serum**	Serana	S-FBS-CO-015
**Poloxamer 188**	Merck	P5556
**Retinoic acid**	Merck	R-2625
**Antibiotic-antimycotic 100X**	Merck	A5955
**Tissue-Tek O.C.T. Compound**	Sakura Finetek	4583
**Vectashield Antifade Mounting Medium**	Vector Laboratories	H-1800-10
**Papain Dissociation kit**	Worthington	I-LK 03150
**RIPA buffer**	Merck	R0278
**cOmplete™, Mini, EDTA-free Protease Inhibitor Cocktail**	Merck	11836170001
**Clarity Western ECL Substrate**	Bio-Rad	1705060
**Pierce BCA Protein Assay kit**	ThermoScientific	23227
**Dithiothreitol (DTT)**	Merck	10197777001
**4x Laemmli sample buffer**	Bio-Rad	1610747
**4-20% Mini-PROTEAN TGX Precast Protein Gels**	Bio-Rad	4561094
**PVDF membrane**	ThermoScientific	10600023

**TWEEN 20**	Merck	P6585

Table S4: List of antibodies used in this study.

Antibody	Dilution	Source	Identifier
**Anti-usherin (C-terminal)**	1:2500	Gift by Dr. Yang [36]	NA
**Anti-ADGRV1**	1:7500	Gift by Dr. Yang [36]	NA
**Anti-whirlin**	1:400 (IHC)<br>1:1000 (WB)	Proteintech	25881-1-AP
**Anti-Rhodopsin**	1:500	Millipore	MAB5356
**Anti-GFAP**	1:400 (IHC)<br>1:1000 (WB)	Dako	Z0334
**Anti-ROM1**	1:400	Proteintech	21984-1-AP
**Anti-ARL13B**	1:400	Proteintech	17711-1-AP
**Anti-F-actin**	1:300	Thermo Fisher Scientific	r-415
**Anti-CD44**	1:300	Thermo Fisher Scientific	MA4400
**Anti-GAPDH**	1:1000	Proteintech	60004-1-lg
**Goat anti-rabbit IgG (H+L) Highly Cross-Adsorbed Secondary Antibody, Alexa Fluor 488**	1:1000	Invitrogen	A-11034
**Goat anti-mouse IgG H&L, Alexa Fluor 555**	1:1000	Abcam	ab150118

**Biotin-SP-Conjugated Affinipure Goat Anti Rat IgG (H+L) + Dylight™ 649-conjugated streptavidin**	1:1000	112-065-003 + 016-490-084	Jackson ImmunoResearch
**Anti-Mouse-IgGκ BP-HRP**	1:5000	SantaCruz Biotechnology	sc-516102
**Anti-rabbit-IgG-HRP**	1:5000	SantaCruz Biotechnology	sc-2357

Product information about the antibodies used in immunohistochemistry (IHC) and Western Blot (WB) analyses.

Supplementary Methods

1. *Colony selection after genome editing*

The day after electroporation, wells were refreshed with 1 mL of mTeSR Plus + CloneR. An additional 250 µL of mTeSR Plus + CloneR (STEMCELL Technologies, Cologne, Germany) was added on day 3. Until day 6, the medium was replaced every day with mTeSR Plus. On day 7, hiPSCs were harvested with GCDR (STEMCELL Technologies, Cologne, Germany), filtered through a 40 µm cell strainer and centrifuged at 1100 rpm for 3 minutes. Cells were counted manually, and 1000 cells were seeded on two Matrigel-coated 10 cm dishes (Corning, Glendale, AZ, USA) with mTeSR Plus + CloneR. Leftover cells were frozen. After two days, a fresh 10 mL of mTeSR Plus medium + CloneR was added to each dish, followed by an additional 2.5 mL on day 10. After this time point, the culture medium was only mTeSR Plus. At day 17, the medium was removed from the dishes, and cells were treated with GCDR at RT. After 4 minutes, GCDR was removed and 5 mL of mTeSR Plus was added. Colonies grown on the dishes were manually scraped off using a p200 pipette tip under a microscope. Each colony was first transferred to a Matrigel-coated 96-well plate containing 150 µL of mTeSR Plus + Fasudil HCl (Focus Biomolecules, Plymouth Meeting, PA, USA). Then, it was broken up by pipetting up and down, and 50 µL of the cell suspension was transferred to a second Matrigel-coated 96-well plate, already containing 50 µL of mTeSR Plus + Fasudil HCl. The second plate was used for screening purposes. In fact, at day 19, DNA was extracted from each well using the QuickExtract DNA Extraction Solution (Merck, Schiphol-Rijk, The Netherlands), according to the manufacturer's instructions. The DNA of each colony was therefore analyzed by digestion with the ScaI restriction enzyme (ThermoFisher Scientific, Waltham, MA, USA), and if the presence of the mutation was confirmed, colonies were amplified and frozen in Cryostor (STEMCELL Technologies, Cologne, Germany). Screening for CNVs was performed using the iCS-digital PSC test, provided as a service by Stem Genomics. Off-

target sites were predicted by IDT software (https://eu.idtdna.com/, access date August 2022). Materials are described in Table S3. hiPSC subclones were tested for the presence of mycoplasma before cryopreservation of the working stocks and prior to differentiation into retinal organoids. Cultures were visually assessed to confirm the absence of bacterial contamination.

2. *Karyotyping analysis*

After cell harvesting, GTG-banded (G-banding with Giemsa and trypsin) metaphases were used for karyotyping at Section Genome Diagnostics, Clinical Genetics, LUMC. Karyotyping was classified as successful and reliable when at least 20 cells in metaphase were available for examination. The regulations of the ISCN (International System for Human Cytogenomic Nomenclature, 2024) were used for describing the karyotype. For analysis and visualization of the chromosomes, the Leica Biosystems CytoVision was used.

3. *Retinal organoid differentiation: media composition*

NIM1 is prepared by adding to DMEM/F12 (1:1): N2 supplement 1X, MEM NEAA 1X (ThermoFisher Scientific, Waltham, MA, USA), Heparin 2 μg/μL, and antibiotic-antimycotic 1X (Merck, Schiphol-Rijk, The Netherlands). NIM2 is prepared by mixing DMEM/F12 (1:1) and DMEM (1X) + GlutaMAX (final concentration 3:1) (ThermoFisher Scientific, Waltham, MA, USA) supplemented with Neurocult SM1 1X (STEMCELL Technologies, Cologne, Germany), MEM NEAA 1X, antibiotic-antimycotic 1X, and 1% Poloxamer (Merck, Schiphol-Rijk, The Netherlands). RLM1 is prepared by mixing DMEM/F12 (1:1) and DMEM (1X) + GlutaMAX (final concentration 3:1) supplemented with Neurocult SM1 1X, MEM NEAA 1X, antibiotic-antimycotic 1X, 1% Poloxamer, 10% FBS (Serana, Brandenburg, Germany), and 100 μM Taurine (Merck, Schiphol-Rijk, The Netherlands). RLM2 is prepared by mixing DMEM/F12 (1:1) and DMEM (1X) + GlutaMAX

(final concentration 3:1) supplemented with N2 1X, MEM NEAA 1X, antibiotic-antimycotic 1X, 1% Poloxamer, 10% FBS, and 100 μM Taurine.

4. *Immunohistochemical analysis*

The organoid sections were blocked for 1 hour at RT with a pre-incubation buffer composed of 10% normal goat serum (ThermoFisher Scientific, Waltham, MA, USA), 0.4% Triton X-100, and 1% bovine serum albumin in PBS. Slides were then incubated with the primary antibodies at 4°C overnight with 0.3% normal goat serum, 0.4% Triton X-100, and 1% bovine serum albumin in PBS. After two washes in PBS for 15 minutes each, slides were incubated for 1 hour at RT with fluorescent-labeled secondary antibodies in 0.1% normal goat serum in PBS. Once the excess of antibodies was removed by two further cycles of washes in PBS, the slides were mounted using the Vectashield Antifade Mounting Medium containing DAPI (Vector Laboratories, Newark, CA, USA), to counterstain nuclei. Sections were imaged on a Leica TCS SP8 confocal microscope (Leica Microsystems, Wetzlar, Germany) using the Leica Application suite X (v3.7.0.20979).

5. *Transmission electron microscopy analysis*

Samples were fixed in 1.5% glutaraldehyde in 0.1M cacodylate buffer at RT by adding double-concentrated fixative to the medium at RT for 1 hour. Samples were rinsed three times in 0.1 M cacodylate buffer and post-fixed in 1%OsO_4/1.5% potassium ferricyanide in 0.1M cacodylate buffer (1 hour on ice). Samples were rinsed again in 0.1M cacodylate buffer three times and dehydrated in a series of ethanol, followed by a series of acetone with EPON (LX112, Ladd research industries, Essex Junction, VT, USA) and finally a step in 100% EPON. Organoids were put in a mold, which was filled up with EPON and polymerized at 70°C over two days. Ultrathin sections (90 nm) were created on a Reichert Ultracut S (Leica Microsystems, Wetzlar, Germany) and, after staining with uranylacetate and lead citrate, examined on an electron microscope (microscope:

Thermofisher (formerly FEI) Tecnai T12 twin (ThermoFisher Scientific, Waltham, MA, USA; Camera: OneView, Gatan)) operating at 120 kV. Overlapping images were collected and stitched together into separate images as previously described [47].

6. *Retinal organoid dissociation*

Retinal organoids were dissociated following an adapted protocol from the Papain Dissociation kit (Worthington Biochemical Corp., Lakewood, NJ, USA) (see Table S3). Twenty-four hours prior to the procedure, the excess of RPE was carefully cut away from every retinal organoid, which was subsequently mechanically divided into very small pieces with the help of tweezers. Each retinal organoid was incubated at 37 °C for 90 minutes on an orbital shaker set to 90 rpm in the presence of 500 µl of freshly prepared dissociation solution (20 units/ml papain and 0.005% DNase). Then, they were gently triturated 5-6 times using a 1 mL pipettor, making sure to avoid the formation of bubbles. The 48-well plate was placed back in the incubator for 10 minutes, after which the organoids were further triturated until obtaining a single-cell suspension. This was added to a tube containing 1 mL of albumin ovomucoid protease inhibitor solution with DNase. Each well was further washed with an extra 1 mL of solution. Tubes were centrifuged at 300x g for 5 min. After removing the supernatant, the cells were resuspended in PBS with 2% BSA/0.01% TWEEN 20 and filtered through a 40 µm cell strainer to remove cell aggregates. A viable cell count was performed. One million cells per sample were resuspended in 100 µl of staining buffer (2% BSA/0.01 % TWEEN 20, PBS) in low bind tubes, with 10 µl of Fc blocking reagent (FcX) (BioLegend Europe, Amsterdam, The Netherlands) for 10 minutes on ice. Then, 0.5 µg of unique cell hashing antibodies were added to each tube and incubated for 20 minutes on ice. After three washing steps with 1 mL of staining buffer, the stained cells were centrifuged at 4 ˚°C for 5 minutes at 350g. Concentration and cell viability were assessed for each sample (Bio-Rad, TC20). After counting, all

samples were merged and resuspended at the desired concentration for single-cell sequencing. A total of 14 organoids were analyzed in a single run and distributed as follows: 4 organoids from the ISO-CTRL line, 4 organoids from the 13KO E11 line, 3 organoids from the 13KO G8 line, and 3 organoids from the 13KO B7 line.

7. *Single-cell RNA sequencing—Computational analysis*

Single cells were demultiplexed based on their HTO enrichment to assign each of them to their sample origins. Barcodes that were positive for only one HTO were classified as singlets and selected for downstream analyses. In the quality control procedure, singlets were filtered in order to keep only cells with nFeature_RNA between 500 and 7000, nCount_ RNA < 30000, and percent.mt <12. The normalization method used was "LogNormalize", with scale.factor = 30000. The FindVariableFeatures function identified the top 2000 genes exhibiting high cell-to-cell variation in the dataset. Principal component analysis (PCA) was then performed on the scaled data. The first 15 PCs captured the majority of the signal and were used to cluster the cells. A total of 14 clusters were identified and visualized on a two-dimensional plot using the Uniform Manifold Approximation and Projection (UMAP) algorithm. The cluster identity was assigned comparing the top markers resulting from the FindAllMarkers function with the well-known retinal-cell-type-specific markers. For downstream analysis, data were subset per cluster and per condition. Then, differentially expressed genes were retrieved using the FindMarkers function (Wilcoxon rank-sum test). Genes with adjusted p-value < 0.05 were used as a query for pathway enrichment analysis via the gProfiler package. Only the terms recurring in at least two subclones were included in the barplots and were attributed the lowest adjusted p-value among the two subclones, unless otherwise specified. In the dot plots, the dot size indicates the percentage of cells

within each cluster expressing the gene of interest, while the color gradient reflects the average expression level of the gene among those cells.

8. *Western Blot analysis*

To obtain tissue lysates, each retinal organoid was incubated on ice with 30 µL of RIPA buffer, prepared by dissolving one tablet of complete proteinase inhibitor (Merck, Schiphol-Rijk, The Netherlands) in 10 mL of RIPA. The organoids were homogenized by use of a BD u-100 insulin needle for about 10 times and left on a shaker for 30 minutes. After spinning them down at 4 °C for 5 minutes at 10000 rcf, the supernatants were transferred to new tubes. Subsequently, 2 µL of sample/BCA protein standard was pipetted in a flat-bottom 96-well plate, along will 200 µL of Thermo Pierce BCA Protein Assay reagents A and B mixed 50:1 (ThermoFisher Scientific, Waltham, MA, USA). The plate was incubated at 37 °C for 30 minutes, after which the BCA absorbance at 562 nm was read. The concentration and final loading volume were calculated for each sample. The lysate was mixed with 1M Dithiothreitol (DTT) (Merck, Schiphol-Rijk, The Netherlands) and 4x Laemmli sample buffer 1:20 (Bio-Rad, Hercules, CA, USA). Samples were boiled at 95 °C for 5 minutes and run on a Mini-PROTEAN TGX Precast gel (Bio-Rad, Hercules, CA, USA) at 50V for 30 minutes and then at 150V for other 30 minutes. Proteins were transferred from the gel to a polyvinylidene fluoride (PVDF) membrane activated in methanol for 5 minutes by assembling them in a cassette soaked with transfer buffer and setting up a run at 30V at 4 °C overnight in a Bio-Rad electrophoresis tank. Transfer buffer 10X was prepared with 30.3 g of Tris base, 144 g of glycine, and milliQ water up to 1L. The working concentration was obtained by mixing 100 mL of the transfer buffer 10X with 150 mL of methanol and 750 mL of milliQ. The membrane was blocked with 5 mL of 5% milk in PBS for 1 hour. Then, it was incubated overnight on a roller at 4 °C with the primary antibody against whirlin diluted 1:1000 in 5% milk in PBS-TWEEN 20. After a wash

with PBS-TWEEN 20 for 15 minutes at RT, the secondary antibody diluted 1:5000 in 5% milk in PBS-TWEEN 20 was added for 1 hour at RT. One quick wash with PBS-TWEEN 20 and one wash for at least 30 minutes reduced the background signal. The membrane was covered with Clarity Western ECL substrate (Bio-Rad, Hercules, CA, USA) for 2 minutes and imaged using Bio-Rad Chemidoc. To perform further analysis with a primary antibody against GAPDH (glyceraldehyde 3-phosphate dehydrogenase), the membrane was stripped with a buffer made of 5 g of glicine, 1 g of SDS, 10 mL of Tween 20, and 800 mL of distilled water, adjusted to a pH of 2.2 with 37% HCl with final volume of 1L. Incubation with fresh stripping buffer was performed at RT twice for 10 minutes. Then, the membrane was washed with PBS for 10 minutes and with PBS-Tween 20 for 5 minutes, after which it was ready to be blocked again. Detailed information on the materials used in this analysis can be found in Table S3 and Table S4. After image acquisition via Bio-Rad Chemidoc under non-saturing conditions, densitometric analysis was performed using Fiji ImageJ version 2.17. Identical rectangles were drawn around each band to obtain their intensities. The local background signal was subtracted prior to quantification. Target protein intensity was normalized to the housekeeping GAPDH protein level to control for loading variability.
